# Supplementary material for: Conductance of porous media depends on external electric fields
Source: Biophys J. 2021 Feb 18;120(8):1431–42. doi: 10.1016/j.bpj.2021.02.012 (PMC8105728; doi:10.1016/j.bpj.2021.02.012)
Supplement: Document S1. Figs. S1–S4 [file mmc1.pdf]

Biophysical Journal, Volume *120*

Supplemental information

Conductance of porous media depends on external electric fields

Leonid P. Savtchenko, Kaiyu Zheng, and Dmitri A. Rusakov

SUPPLEMENTARY FIGURES

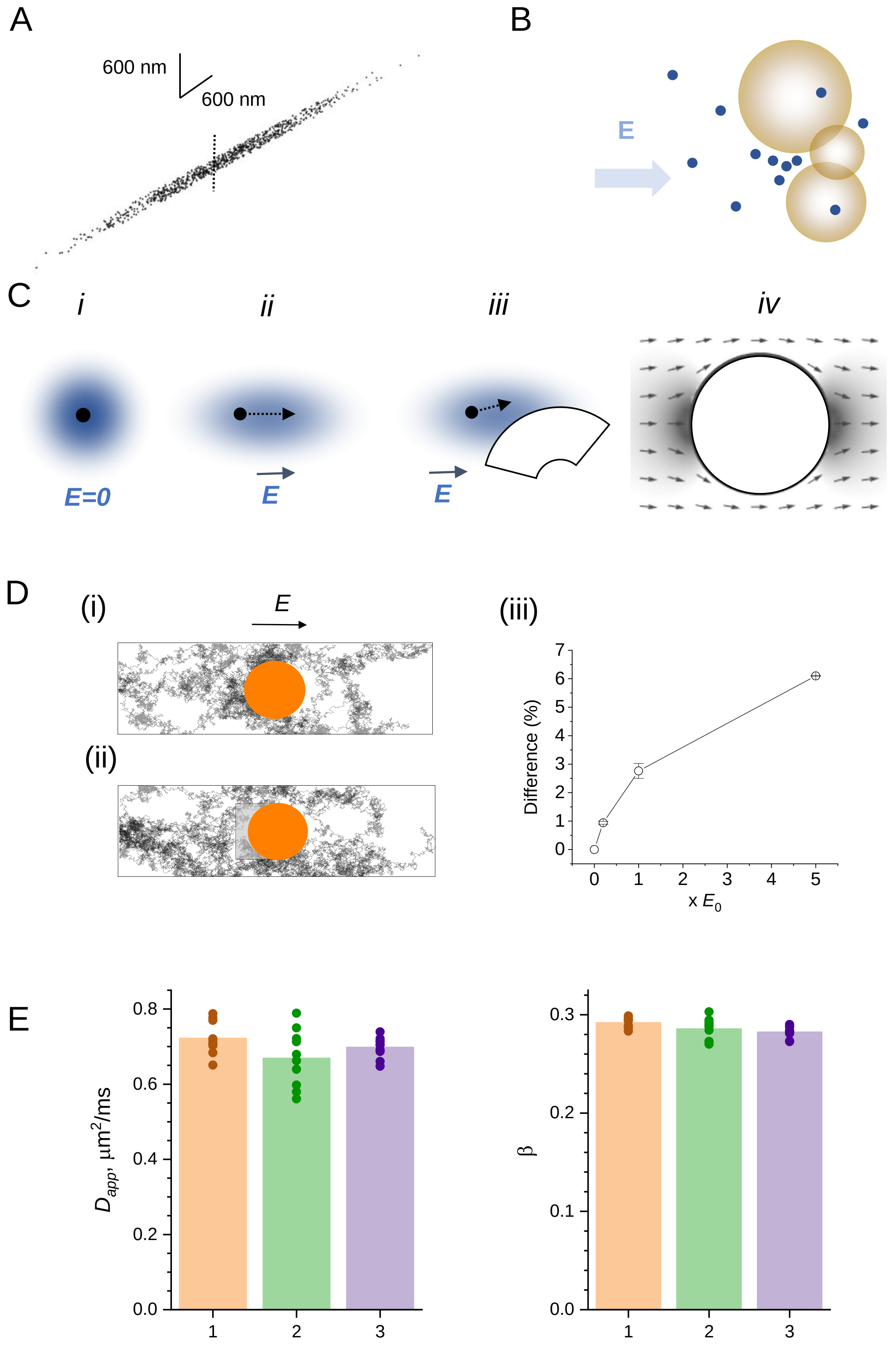

Supplementary Figure S1. MC simulations of particle diffusion under uniform electric filed.

(A) Simulation example: snapshot of the scatter of diffusing particles (dots) along a 0.1 µm wide - 10 µm long cylinder, 0.1 ms post-release from the centroid (dotted line); space fraction occupied by spherical obstacles (3-33 nm diameter, not shown) is β = 0.2, no electric field (*E* = 0); actual simulation data rendered by OriginPro.

(B) A schematic illustrating the possibility of 'dead-ends' occurring in the scatter of overlapping spheres (orange circles) that might trap molecules (blue dots) diffusing under field *E* > 0 (blue arrow).

(C) Schematic illustrating (not to scale), average behaviour of a Brownian particle; blue shade, probability density of particle occurrence (dot, starting point), under no electric field (*i*), under uniform field *E* (*ii*; dotted arrow, average drift), and facing a spherical obstacle barrier (*iii*); the latter scenario is similar to the local direction of electric field (*iv*; small arrows, standard example of field deviation near a quasi-dielectric sphere under uniform external field in a high-permittivity medium; grey shadow, relative field strength; (https://demonstrations.wolfram.com/DielectricSphereInAUniformElectricField/,polarisation effects are ignored), or otherwise the directional vector field of a hydrodynamic; flow near a round obstacle.

(D) MC simulation test ('flat' 1D diffusion) illustrating that electric field distortion near a circular obstacle (i.e., partial shielding from field) has little impact on the calculated diffusivity under a homogenous electric field: (*i*) trajectory snapshot of particle diffusion (n = 10 particles / trials, starting point at the left-boundary midpoint) around a circular obstacle, under field $E = 5E_0$; (*ii*) Example as in (*i*), but with a field-shielding area (grey shade) extending to ~1.3 of the obstacle radius, where *E* = 0: in a simple case of dielectric spheres in a uniform electric field, the field configuration at >1.3 radius from the sphere midpoint approaches the uniform field away from the sphere, across a wide range of dielectric constants (e.g., https://demonstrations.wolfram.com /DielectricSphereInAUniformElectricField/); (*iii*) Summary, the percentage difference (mean ± SEM, n = 10) between D_{app} values calculated for cases shown in (*i*) and (*ii*), for varied values of *E*. Key model parameters: circle radius 30 nm, 'flat tube' width 100 nm, $E_0 = 10^4$ V/m, $D_{free} = 1$ µm^2 ms^{-1}.

(E) MC simulation test, illustrating estimation of the average diffusivity (left graph, 1D case, n = 10 simulation runs) for three different arrangement of spherical obstacles under similar β ~ 0.29 (mean ± SD): (1) 8000 non-overlapping spheres, radius 10 nm (D_{app} = 0.722 ± 0.043 µm^2/ms); (2) overlapping spheres with the radius distributed evenly between 3-30 nm (D_{app} = 0.669 ± 0.076 µm^2/ms); and (3) 3500 non-overlapping spheres, radius of 14.2 nm (D_{app} = 0.698 ± 0.028 µm^2/ms); dots, individual MC simulation runs. MC simulations for *β* ~ 0.4 gave D_{app} = 0.55 ± 0.13 µm^2/ms and 0.51 ± 0.13 (mean ± SD; n = 10 runs) for cases (1) and (2), respectively.

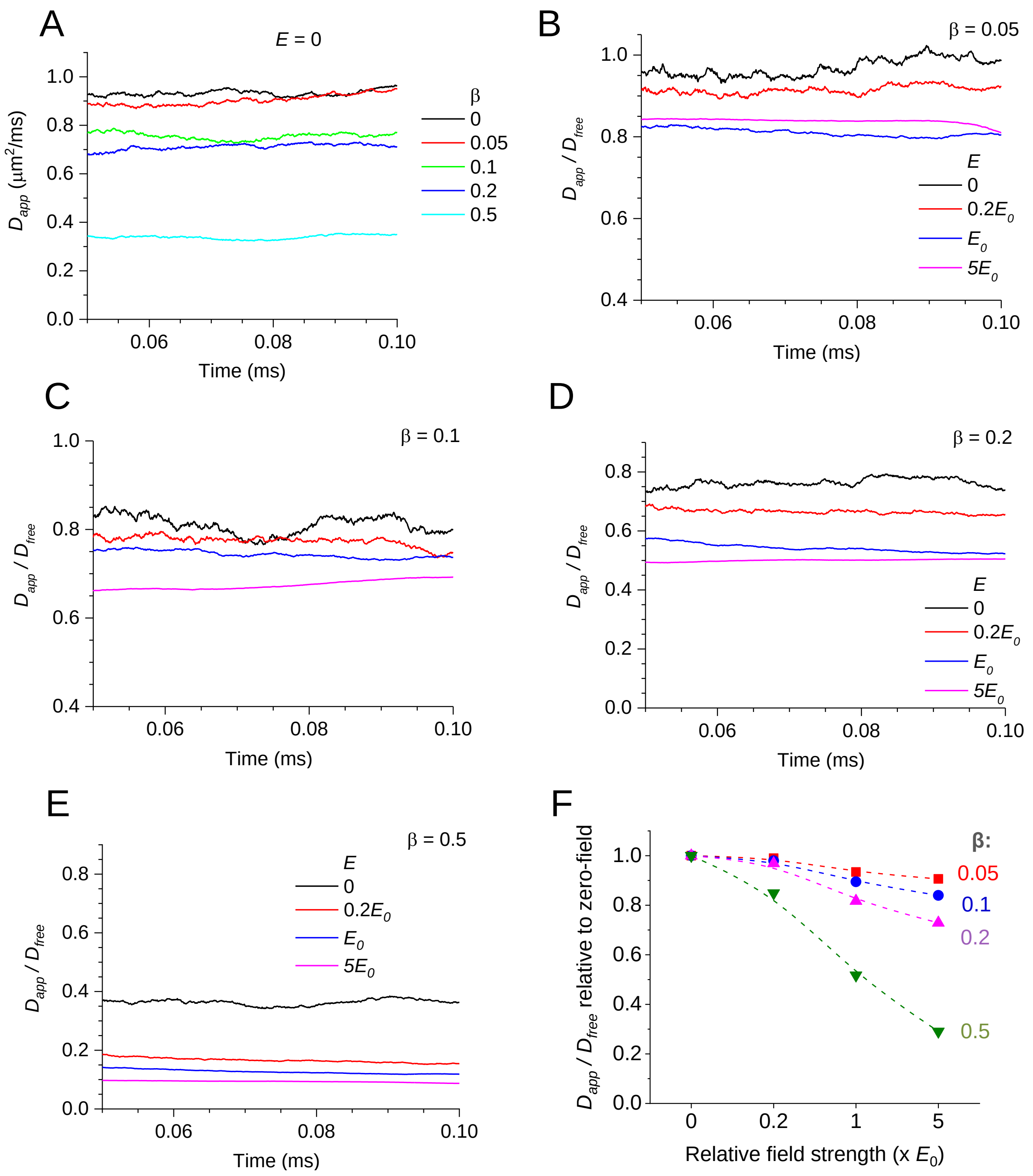

Supplementary Figure S2. MC simulations estimating apparent permeability of obstacle-filled medium in 1D electric field.

(A) Example of a MC simulation run: time course of apparent diffusivity D_{app} (along the cylinder as) under E = 0 (repeat of the test shown in Fig. 1B).

(B-E) Examples of MC simulation runs: time course of medium permeability $\theta = D_{app} / D_{free}$ (narrow cylinder), estimated for different values of β and E as indicated ($E_0 = 10^4$ V/m).

(F) A summary of MC simulations showing the dependence between field strength and permeability θ (normalised to the value at E = 0), under 1D uniform electric field, for several β values, as indicated**.**

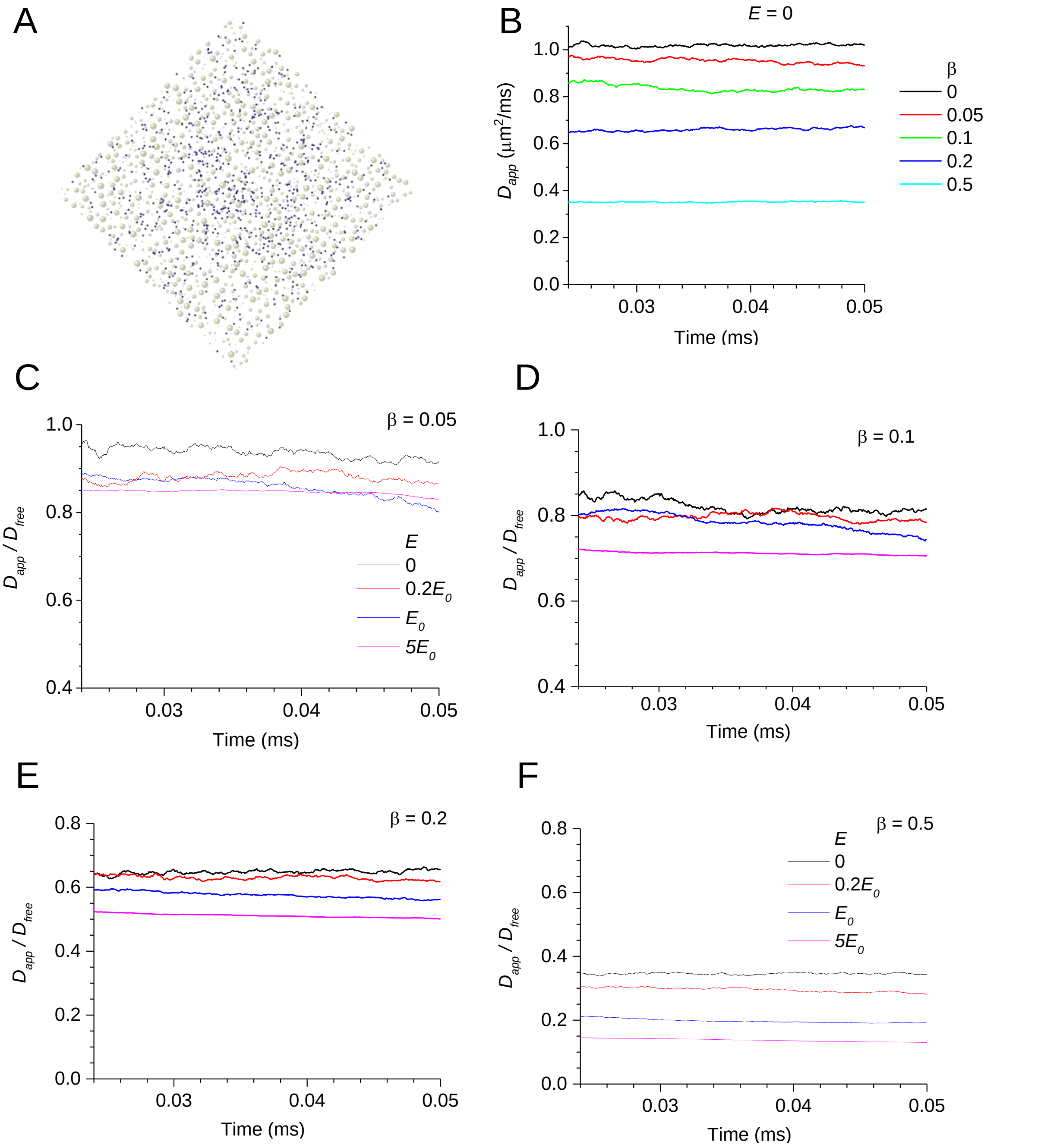

Supplementary Figure S3. Estimation of medium permeability for ions in a flat obstacle-filled cleft under a 2D radial field.

(A) Simulation example: snapshot of the scatter of diffusing particles (dots) in a flat 20 nm wide cleft along, 0.1 ms post-release from the centre; as in Fig. 2A (4 x 4 µm arena), shown as a 2D projection, for clarify. Other notations are as in Fig. 2A.

(B) Example of a MC simulation run: time course of apparent diffusivity D_{app} in the flat cleft under E = 0.

(C-F) Examples of MC simulation runs: time course of medium permeability $\theta = D_{app} / D_{free}$, estimated for different values of β and E as indicated ($E_0 = 10^4$ V/m).

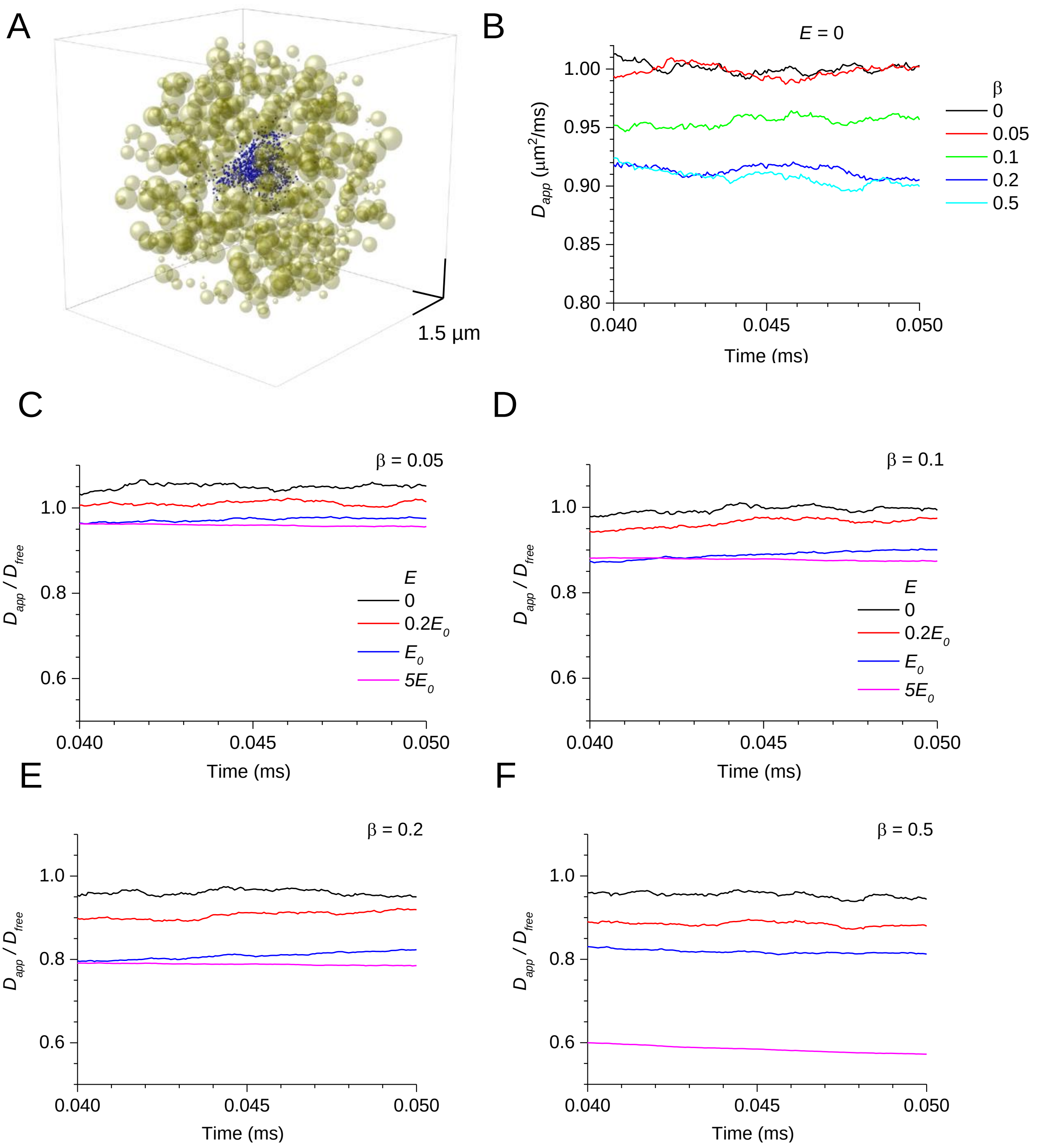

Supplementary Figure S4. Estimation of medium permeability for ions diffusing in space under 3D radial field.

(A) Simulation example: a scatter of diffusing particles as in Fig. 2D text, shown here on a larger scale for illustration purposes. Other notations are as in Fig. 2D.

(B) Example of a MC simulation run: time course of apparent diffusivity D_{app} in 3D space under $E = 0$.

(C-F) Examples of MC simulation runs: time course of medium permeability $\theta = D_{app} / D_{free}$, estimated for different values of β and E as indicated (same notations throughout; $E_0 = 10^4$ V/m).
