# Supplementary material for: Conductance of porous media depends on external electric fields
Source: Biophys J. 2021 Feb 18;120(8):1431–42. doi: 10.1016/j.bpj.2021.02.012 (PMC8105728; doi:10.1016/j.bpj.2021.02.012)
Supplement: Document S2. Article plus supporting material [file mmc2.pdf]

Biophysical Journal
Article

Conductance of porous media depends on external electric fields

Leonid P. Savtchenko,[1,*] Kaiyu Zheng,[1] and Dmitri A. Rusakov[1,*]
[1]UCL Queen Square Institute of Neurology, University College London, London, United Kingdom

ABSTRACT In obstacle-filled media, such as extracellular or intracellular lumen of brain tissue, effective ion-diffusion permeability is a key determinant of electrogenic reactions. Although this diffusion permeability is thought to depend entirely on structural features of the medium, such as porosity and tortuosity, brain tissue shows prominent nonohmic properties, the origins of which remain poorly understood. Here, we explore Monte Carlo simulations of ion diffusion in a space filled with overlapping spheres to predict that diffusion permeability of such media decreases with stronger external electric fields. This dependence increases with lower medium porosity while decreasing with radial (two-dimensional or three-dimensional) compared with homogenous (one-dimensional) fields. We test our predictions empirically in an electrolyte chamber filled with microscopic glass spheres and find good correspondence with our predictions. A theoretical insight relates this phenomenon to a disproportionately increased dwell time of diffusing ions at potential barriers (or traps) representing geometric obstacles when the field strength increases. The dependence of medium ion-diffusion permeability on electric field could be important for understanding conductivity properties of porous materials, in particular for the accurate interpretation of electric activity recordings in brain tissue.

SIGNIFICANCE From nanomaterials to animal tissues, diffusion permeability of porous media has been attributed entirely to their structure, which is often summarized as porosity and tortuosity. Here, we simulate a sphere-filled space to find that its conductance decreases with stronger electric fields and confirm this in a physical experiment with a theoretical treatment highlighting its key parameters. This finding has some potentially fundamental implications for our understanding of electricity in porous media. For instance, it suggests that routinely recorded brain field potentials may not necessarily scale linearly with the strength of current sources inside the brain tissue. On the microscopic scale, it predicts retarded diffusion of charged molecules, heterogeneous charge accumulation, and possibly supralinear heat dissipation with increased electrical activity.

INTRODUCTION

Physiological signaling in animal tissue relies on rapid diffusion of electrolyte ions in the extracellular (or intracellular) space filled with relatively immobile obstacles, from macromolecules to microscopic cellular structures. Compared with a free medium, diffusion in obstacle-filled or porous media is retarded. According to the classical Nernst-Einstein equation, electrolyte conductivity G scales with ion diffusivity so that

$$G = \frac{F^2}{RT}\sum_{n=1}^{i} D_n q_n^2 C_n, \quad (1)$$

where q_n, C_n, and D_n are, respectively, the valence, concentration, and diffusivity of the nth ion species, F is Faraday's constant, R is gas constant, and T is absolute temperature (Table 1). Thus, at constant ion concentrations, G is proportional to diffusivity (which may not be the case when ion concentrations vary or are spatially nonuniform). Generally, retardation of ion diffusion by obstacles corresponds to increased electrical resistance, which in turn affects the scale and dynamics of electric events critical for cell function, particularly in the brain, both on the scale of tissue (1) and the nanoscale (2). Numerous studies have focused on the brain extracellular space to establish that its net diffusion retardation effect is determined by the medium porosity α (space volume fraction available for diffusion) and tortuosity λ, which corresponds to the apparent diffusion coefficient $D_{app} = D_{free}/\lambda^2$ (D_{free} is free-medium diffusivity) (1,3) or otherwise diffusion permeability $\theta = D_{app}/D_{free} = \lambda^{-2}$ (4). The values of α, λ, or θ in brain tissue have been

Submitted April 1, 2020, and accepted for publication February 1, 2021.
*Correspondence: leonid.savtchenko@ucl.ac.uk or d.rusakov@ucl.ac.uk
Editor: Valentin Nagerl.
https://doi.org/10.1016/j.bpj.2021.02.012
© 2021 Biophysical Society.
This is an open access article under the CC BY license (http://creativecommons.org/licenses/by/4.0/).

TABLE 1 Key notations and parameters used

Notation	Meaning/Parameter	Value
k	Boltzmann constant	1.38×10^{-23} m^2 kg s^{-2} K^{-1}
T	Absolute temperature	293 K
R	Gas constant	8.31 kg m^2 K^{-1} mol^{-1}s^{-2}
F	Faraday constant	96,485 C/mol
e	Elementary charge	1.6×10^{-19} C
C	Concentration	theory
D	Diffusion coefficient	theory
q	Valence	theory
D_{free}	Free-medium diffusivity	1 μm^2/ms
D_{app}	Apparent diffusion coefficient	varied
$\theta = D_{app}/D_{free}$	Medium permeability	varied
θ_0	Medium permeability at $E = 0$	varied
$\theta_{\beta\sim0.3}$	Medium permeability at $\beta = 0.3$	varied
E_0	Reference electric field strength	10^4 V/m
E	Electric field strength	$0.2E_0$–$5E_0$
$D_{E=0}$	Diffusion coefficient at $E = 0$	varied
N	Number of diffusing particles	2,000 (200 for test runs)
β	Sphere-occupied volume fraction	0–0.5
-	Number of spheres	200–15,000
-	Sphere diameter	3–33 nm
$\alpha = 1-\beta$	Medium porosity	0.5–1
$\kappa = RT/F$	Constant	theory
τ_-	Time lost to navigate barrier	theory
τ_+	Time gained behind barrier	theory
H	Height of potential barrier	theory
-	Electrolyte cell LHW	10 mm × 10 mm × 2 mm
-	Electrolyte cell temperature	23–24°C

evaluated on the scale of several microns and above (3,5,6). Recently, advances in live super-resolution imaging have extended this quest to the nanoscale structures (7,8), whereas molecular mobility in the extracellular and intracellular lumen on the nanoscale has been evaluated using time-resolved anisotropy fluorescence lifetime imaging microscopy (9). In this context, it has routinely been assumed that the architectural features of porous media (such as λ, θ, and α, or more complex shape variables) principally define their effective conductivity (10,11).

When the electric field is present, ion movement follows the classical Nernst-Planck (NP) relationship

$$\frac{dC}{dt} = -\nabla(J_D + J_E), \tag{2}$$

where C is ion concentration, $J_D = -D\nabla C$ and $J_E = -Dq(F/RT)C\nabla V$ are, respectively, diffusion and electric drift (migration) components, V is field potential (gradient ∇V is equivalent to field strength E). Eqs. 1 and 2 are key to the traditional interpretation of electrophysiological recordings; it assumes constant electrolyte conductivity G, which depends simply on the bulk concentration of ions. Indeed, in the case of the mammalian brain, the common assumption has been that the NP formulism of electrodiffusion applies to the tortuous extracellular space with diffusivity corrected for θ (12–14).

More recently, however, it has been shown that brain tissue shows prominent nonohmic properties that might affect interpretation of local field potentials (15–18), whereas physical tests in porous ceramics indicated that permittivity of such media could depend on the electric field strength (19). There has been a significant progress in advancing theoretical work that focuses on electrodiffusion in porous media (e.g., discussed in (20,21)), including brain tissue (22). On the nanoscale, anomalous diffusion arising due to diffusion obstacles and electric field interactions in cell membranes and neuronal dendrites has also been explored (23,24). However, the influence of external electric fields on ion-diffusion permeability of such obstacle-filled media remains poorly understood.

Here, we focus on this particular issue as a first principle, without attempting to address or generalize evaluation of apparent diffusivity or permittivity for porous media of various types. To understand the basic phenomenon, we therefore explore Monte Carlo (MC) simulations mimicking electrodiffusion of charged particles across the space filled, to a varied degree, with inert, partly overlapped spherical obstacles. Although not critical for establishing theoretical principles, our simulation parameters were chosen to roughly replicate the movement of small ions on the microscopic scale in the interstitial (or intracellular) brain tissue lumen filled with macromolecules and other nanoscopic obstacles. Our simulations predict that increasing the external field strength lowers porous-medium diffusion permeability, thus deviating from the classical NP theory. We test this prediction by using voltammetry measurements in an electrolyte filled with nonpolarizing dielectric (glass), microscopic spheres, and suggest a first-approximation theoretical insight into the underlying principles.

MATERIALS AND METHODS

MC simulations: electrodiffusion

MC simulation algorithms were designed and run using MATLAB (The MathWorks, Natick, MA), based on the classical NP relationship (Eq. 2), as detailed and tested against experimental recordings earlier (25,26). The standard random walk procedure for individual particles (ions) was thus implemented in which the electric field drift (migration) was calculated from the particle speed as $(dr/dt) = -\mu\mathbf{E}$, where mobility $\mu = Dq(F/RT)$, vector $\mathbf{E} = \nabla V$ is the field strength, and $\mathbf{r}$ is the coordinate vector (r is radial coordinate). Thus, ion particles positioned at time t at point $\mathbf{r}(x, y, z)$ were moved to point $\mathbf{r}_1(x_1, y_1, z_1)$ over time increment Δt so that in the case of the one-dimensional (1D) field $x_1 = x + \Delta x \times \delta + \Delta t\frac{eEDx}{kTr}$ with $E = 0$ for particle displacement into y and z directions, Δx corresponds to the mean-square displacement in the Einstein's diffusion equation for 1D Brownian motion $\Delta x^2 = 2D\Delta t$, δ denotes a "delta-correlated" (independently seeded and uncorrelated) uniform random number from the $(-1, 1)$ range to reflect that Brownian particles are equally likely to move into either direction, e is elementary charge, and k is the Boltzmann constant (Table 1). In the case of the two-dimensional (2D) radial field (inside a flat and narrow 2D slab), this expression had $E = 0$ for z direction; in the three-dimensional (3D) radial

field (3D open space), all directions had the field term. As the drift component was added at every diffusion step, the diffusion coefficient calculation was dealing with time and distance in a fixed coordinate system.

To avoid occasional numerical deadlocks for particles trapped near the space dead-ends formed by aggregated overlapped spheres (Fig. S1 *B*), we implemented the duty-cycle translational movements in a contiguous 3D space over all directions rather than over the rectangular 3D-lattice vertices used by us and many others previously. The duty-cycle time step Δt (usually <0.1 μs) was set to be small enough to prevent particles from "tunnelling" through the smallest 6-nm-wide obstacles.

The basic "reference value" field strength E_0 was set at 10^4 V/m, which roughly corresponds to the field generated by a synaptic current of 10–50 pA toward the center of the 10–15-nm-thick, 0.5-μm-wide synaptic cleft, with the medium resistance of $\sim$100 Ohm cm (9,27,28). Accordingly, for stronger electric fields ($E > 2E_0$), we adopted $\Delta t < 0.01$ μs. For the sake of simplicity and to separate the effect of field geometry per se, we have assumed constant field strength E for both uniform (1D) and radial (2D and 3D) fields, whereas under the common 2D and 3D scenarios, field is attenuated with a factor of r^{-1} and r^{-2}, respectively, where r is the distance to the infinitesimal central source. Instead of incorporating these factors in our calculations, we considered the 1D case as the first-principles scenario and addressed the effect of weakening the field separately by exploring E-values over an order of magnitude.

The particle-wall interaction was simulated as an elastic collision, and electrostatic interactions with the obstacle surfaces were ignored on the assumption that the dimensions of the free diffusion space were much larger than the electrolyte double layer.

The initial conditions routinely included 2000 particles injected instantaneously in the arena center (within a 10-nm sphere at the coordinate origin, outside any obstacle). This reflected the case when ions flowing through the open channel generate the field in which they and other ions diffuse, such as inside the synaptic cleft (28). We also ran arena-size trials for each new condition using 200 diffusing particles (see below). Ten full trials with 2000 particles each were carried out for the statistical estimates of average diffusion permeability values, with the sphere distributions generated anew every trial. In addition, we systematically ran single control trials involving 2000 particles to verify the computational stability for the diffusion steps, sphere distributions, and the interactions between particles and obstacles.

MC simulations: sphere-filled space

The main parameter controlling the distribution of spheres is the volume fraction β occupied by them. The β-value was calculated by 1) scattering 10^5 test points uniformly randomly across the entire space, and 2) calculating the proportion of the point falling outside the spheres. We verified that increasing the number of such test points to 10^6 altered β by $<1\%$, suggesting asymptotic accuracy. The space (arena) dimensions were set large enough to reach a stationary value for the apparent diffusion coefficient under the strongest electrical field (fluctuation was less than $\pm1\%$ for the last 20% of the simulated diffusion time). As noted above, the arena size was routinely trialled and established using ad hoc simulations with 200 particles under the strongest tested electric field without spheres.

To fill the space with overlapping spheres that have a distributed size, the following algorithm was adopted. First, we prohibited spheres to occupy a 3-nm-wide space around the coordinate origin where the particles start their random walk. Second, in each duty cycle, we generated a random sphere location (set of coordinates) with the arena space and the random radius value (distributed in accord with the designated density function) for the sphere. Third, we repeated the cycle until β approaches the required value with $\sim$5% accuracy.

To replicate our empirical arrangement with glass spheres, we attempted an algorithm filling the space with equally sized nonoverlapping spheres. Random packing of equal spheres has a theoretical density maximum of $\beta\sim64\%$, assuming that the arena size is much larger than that of spheres (29). In computational practice, however, as the volume fraction β occupied by the simulated spheres reaches $\sim$50%, it becomes progressively difficult to insert further spheres. In the majority of cases, the algorithm stalls because of the lack of available space. It turns out that a similar deadlock occurs when the spheres have a predefined distribution of their size, at least in the case of even distribution. Therefore, we asked whether the cases of overlapping versus nonoverlapping spheres differ significantly in terms of the apparent diffusion coefficient and found little difference, at least for $\beta < 0.5$ (see Fig. S1 *D*). This was consistent with the observation that the overlaps occupied only a small fraction ($<4\%$ for $\beta = 0.5$) of the arena volume, thus suggesting little effect overall, which also applies to enhanced trapping due to the electric field.

The overall size (cutoff distance) of the diffusion arena was determined by the condition that it should be large enough to contain $>99\%$ particles during at least 0.5-ms postrelease in the strongest-field case $E = 5E_0$ and with $\beta = 0$ (free space, no obstacles). At the same time, the arena had to be sufficiently small to allow technically feasible computation times, which increased supralinearly with greater β or with the number of spheres introduced as obstacles.

Computing environment

Simulations were carried out on a dedicated, ad-hoc-built, 8-node BEOWULF-style diskless PC cluster running under the Gentoo LINUX operating system (kernel 4.12.12), an upgraded version of the cluster described earlier (5). Individual nodes comprised an HP ProLiant DL120 G6 Server (Hewlett Packard Enterprise, San Jose, CA) containing a quad-core Intel Xeon X3430 processor and 8 GB of DDR3 RAM (Santa Carla, CA). Nodes were connected through a NetGear Gigabit Ethernet switch to a master computer that distributes programs and collects the results on its hard disk. Optimization and parallelization routines were implemented by AMC Bridge (Waltham, MA).

Calculating the apparent diffusion coefficient

The apparent diffusion coefficient for the particle cohort, averaged over time t in any of the three directions, was calculated as $D_x = (1/2N)\sum_{i=1}^{N}(x_i^2/t)$ (similar for y and z), where N is the number of diffusing particles, and x_i^2 reports the particle's mean-square displacement over time t. The diffusion coefficient values were calculated continuously during simulation runs as long as all original diffusing particles remained within the simulation arena. Normally, once a single particle has left the open boundary of the simulation area, computations stopped.

Analytical solutions of the NP electrodiffusion equations

In analytical estimates of the particle concentration profiles, we solved the NP equation using the built-in MATLAB function *pdepe* (The MathWorks). This function enables solving initial-boundary value problems for the parabolic-elliptic type of partial differential equations.

Conductance measurements

Electrolyte conductance was measured using a classical shunt resistor method (e.g., p. 175 in (30)). Various voltages supplied by a constant voltage source with maximal current limited to 2A (TTi EX4210R 42V 10A model) were applied to a presterilized, industry-standard electroporation chamber (Molecular BioProducts #5520; W $\times$ L $\times$ H: 2 mm $\times$ 10 mm $\times$ 25 mm; Fig. 4 *A*). Because of the large current passing through the circuit at high voltage steps, a shunt resistor of 10 A/V (RS257-391) was connected in series to the chamber from which a small voltage drop across the resistor was measured using a national instrument analog to digital converter (NI BNC-2090). To deal with any resistive heating effects in the chamber under high currents, we applied short (10-ms) voltage pulses with a high current

reed relay (Cynergy3 S8-0504; Wimborne, UK) connected in series before the chamber. The short 10-ms pulse applied to the circuit had no resistive heating effect; the monitored solution temperature in the chamber was 23–24°C throughout tests. Signal timing was gated by a pulse generator (Master-8; AMPI, Jerusalem, Israel), which was triggered from the same national instrument board using acquisition control software WinWCP (University of Strathclyde).

Three different solutions were made: deionized water (H_2O, ELGA PURELAB machine (ELGA LabWater, Woodridge, IL), resistivity 15 MOhm/cm at 25°C;), sodium chloride (NaCl, S7653; Sigma-Aldrich, St. Louis, MO; 153 mM solution in deionized water solution), and ACSF. The ACSF solution composition was as follows: 126 mM NaCl (S7653; Sigma-Aldrich), 2.5 mM KCl (P9333; Sigma-Aldrich), 1.3 mM $MgSO_4 \cdot 7H_2O$ (M1880; Sigma-Aldrich), 1.25 mM NaH_2PO_4 (S0751; Sigma-Aldrich), 10 mM glucose (G8270; Sigma-Aldrich), 26 mM $NaHCO_3$ (S6014; Sigma-Aldrich), and 2 mM $CaCl_2 \cdot 2H_2O$ (C3881; Sigma-Aldrich). The electrolyte strength was thus chosen to be in the physiological range below the levels (>0.2 M) at which, classically, ion-ion interactions could affect conductivity (31).

DC voltages were applied to the opposite electrodes in the chamber (Fig. 4 *A*), and small voltages across the shunt resistor were measured and converted to currents. The procedure was carried out in a free solution and then repeated with 13–45-μm lime soda glass spherical microbeads (Specialty Products 201-002-003; Mo-Sci, Rolla, MO). The beads were slowly (to avoid void formations) loaded into a ~150-μL freshly prepared solution until the chamber was full (Fig. 4 *B*). The electrolyte volume required to fill the chamber volume occupied by the spheres provided an estimated valued of $\beta = 30 \pm 2\%$ (mean ± SEM).

Because electrical conductance depends on the temperature, it was also important to avoid temperature rises. This was achieved by using short 10-ms voltage steps with 60-s resting periods; the solution temperature thus held at 23–24°C. The short electrical pulse also minimizes electrode polarization, which might, in principle, lead to an accumulation of ionic species near the surface, hence unwarranted chemical reactions. Special care was taken to avoid bubble formation and accumulation in the solution during electric pulses: tests were terminated upon detection of any microscopic bubbles (usually after several trials). To avoid such and further time-dependent concomitants of the pulse application, we used only the first 3 ms of the pulse, and no more than five trials per cuvette.

Data availability

The data sets generated and/or analyzed during this study are available from the corresponding authors upon request.

Source code availability

The data sets source codes used in this study are available from the corresponding authors.

RESULTS

Electrodiffusion in uniform 1D electric field

We first asked to what extent geometric obstacles would affect ion movements along a narrow long cylinder (diameter << length), a basic case of 1D diffusion. We note that although in this case the overall diffusion flux is 1D, on the scale compatible with the cylinder diameter, diffusion trajectories and obstacle geometries are essentially 3D. For the sake of simplicity, we set the free-medium diffusion coefficient at $D_{free} = 1\ \mu m^2/ms$, which is typical for small ions in aqueous solutions, and the cylinder diameter at 0.1 μm to roughly represent extra- or intracellular lumen of brain tissue. Zero-size diffusing particles were released in the cylinder centroid and allowed to move freely (Materials and methods; Fig. 1 *A*; Fig. S1 *A*), with the cylinder walls providing a reflecting boundary. To mimic macromolecular hindrance, small spheres with an evenly distributed diameter over the 3–33 nm range were scattered uniformly with overlap throughout the space (Materials and methods). Overlapping spheres could sometimes form dead-ends for diffusing particles (Fig. S1 *B*), reflecting nonconvex geometries of real microscopic obstacles. The MC algorithm incorporating Brownian movement and electric field drift (Materials and methods) stochastically directed the particle flow around obstacles, roughly following the field lines around dielectric spheres (Fig. S1 *C*). Although our simulations adopted a uniform field throughout the space and thus ignored its distortion near dielectric spheres, the comparison of limiting cases (uniform versus zero-field near spheres) indicated that the related diffusivity error was fairly negligible (~6% at the strongest field; Fig. S1 *D*). We have also found that changing the nonoverlapping to overlapping sphere pattern altered D_{app} values only within ~7% at $\beta \sim 0.4$ and within ~4% at $\beta \sim 0.3$ (Fig. S1 *E*), thus projecting a smaller still effect at smaller β.

Equipped with these settings, we sought to test fours scenarios for β taking four values between 0 and 0.5 to reflect diffusion retardation on the nanoscale assessed with time-resolved fluorescence anisotropy imaging (9). The time-averaged apparent diffusion coefficient D_{app} showed significant variability in the initial stages of the test, reflecting anomalous diffusion (Fig. 1 *B*, zero field), as discussed previously (32). Reassuringly, in a free medium, ($\beta = 0$) D_{app} was converging to D_{free} (Fig. 1 *B*, *black dotted line*; Fig. S1 *B*). Simulations also showed that D_{app} decreased monotonously with greater β (Fig. 1 *B*), consistent with earlier assessments of macroscopic diffusion in porous brain tissue (4,33,34).

We next introduced a uniform electric field that was collinear with the cylinder axis (Materials and methods). To mimic the case of an ion channel current that generates voltage gradient for the current carrying and other ions in the nearby lumen, we considered the outward field direction that would force particles away from the diffusion source (Fig. 1 *A*, *arrows*). We explored a range of field strength E centered around $E_0 = 10^4$ V/m, which roughly represents the field generated by a synaptic current carried mainly by Na^+ ions toward the synaptic cleft center at small excitatory synapses (Materials and methods). The simulation outcome was somewhat unexpected. Introducing geometric hindrance retarded particle diffusion to a greater degree under electric field if compared with the zero-field scenario in the same setting (Fig. 1, *B* and *C*). The effect depended monotonously on both E and β (single-run simulation examples in Fig. 1, *C* and *D*; further detail in Fig. S2, *A–E*).

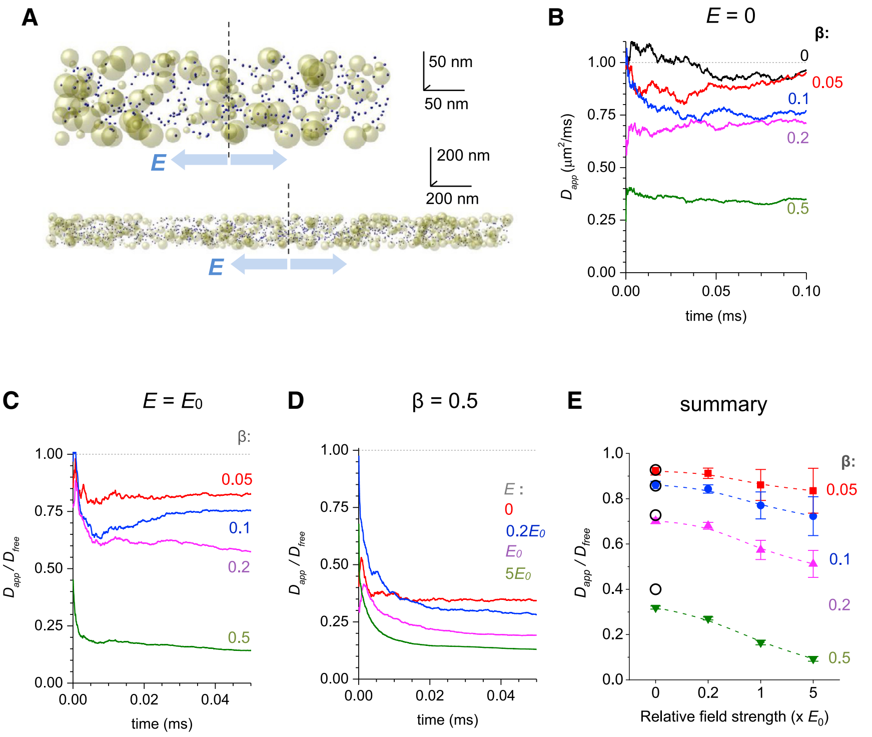

FIGURE 1 Ion diffusion permeability of obstacle-filled media in uniform electric field depends on field strength. (*A*) Snapshot of the simulated scatter of diffusing particles (*blue dots*) in a 0.1-μm-wide, 10-μm-long cylinder (*fragment*; two different scales as shown; actual simulation data rendered by OriginPro) at 0.1 ms postrelease from the centroid (*dotted line*), with $\beta = 0.2$ volume fraction occupied by obstacles (*yellow spheres*, diameter 3–33 nm) and electric field strength $E = 0$; blue arrows indicate the field direction. (*B*) An example of an MC simulation run: time course of apparent diffusivity D_{app} (along the cylinder as in *A*) for different values of β as indicated. (*C*) An example of an MC simulation run: time course of diffusion permeability $\theta = D_{app}/D_{free}$ under electric field $E = E_0$ (10^4 V/m) and over the range of β-values as shown; see Materials and methods for model parameters. (*D*) An example of an MC simulation run: time course of θ for a medium with for $\beta = 0.5$ over the range of E as indicated. (*E*) Statistical summary of simulation experiments depicted in (*B*)–(*D*) (additional examples of individual MC runs are shown in Fig. S2); dots $\pm$ error bars indicate diffusion permeability $\theta = D_{app}/D_{free}$ (mean $\pm$ SD) values for n = 10 runs completed for each set of β- and E-values, as indicated; the overall effect of either factor E (four levels) or β (four levels) on θ is at $p < 0.001$, determined by two-way analysis of variance (ANOVA) ($F_E = 79.0$, $F_\beta = 1366$). To see this figure in color, go online.

Repeating simulations runs 10 times with 2000 particles each for each scenario provided a robust summary (Fig. 1 *E*; Fig. S2 *F*), suggesting that in obstacle-filled media, the relationship between field strength and ion transfer (current) is sublinear. It appears therefore that the obstacles decelerate ion movement to a disproportionately greater degree in stronger fields. Reassuringly, in these simulations, the D_{app}/D_{free} ratios representing diffusion permeability θ were, while under $E = 0$, in good correspondence with the Maxwell's relationship for porous media (35) $D_{app} = \frac{2\alpha D_{free}}{3-\alpha}$, where porosity $\alpha = 1 - \beta$ (Fig. 1 *E*, *open circles*). However, under $E > 0$, our data suggest that porous-medium electric resistance, or ion-diffusion permeability, depends on the field strength rather than on the medium properties alone, thus deviating from the Ohm's law.

Electrodiffusion in radial 2D and 3D electric fields

Molecular diffusion inside narrow 2D clefts with a local current point-source is a common scenario in the brain neuropil where intercellular signal exchange occurs in an electrolyte medium between the opposing cell membranes populated with ion channels. Our next test was therefore to release diffusing particles in the 20-nm-wide (characteristic interstitial width) flat cleft with an accelerating radial field (Fig. 2, *A* and *B*). For simplicity, field strength E was maintained as uniform; we have previously shown that in the 2D synaptic cleft, with a spatially uniform distribution of postsynaptic channels, the field is closer to uniform than to the classical r^{-1} decay for radial field with cylindrical symmetry (r is the distance from the center). Furthermore, when the diffusion distance of interest is much smaller than the distance from the field source, uniform field provides canonical linear approximation. An additional reason for keeping uniform E was to try and separate the effects of field geometry and field strength on diffusion permeability. Other simulation parameters were similar to the 1D case shown above (Fig. 1). Again, the simulation outcome suggested that the electric fields, while accelerating overall diffusion escape of charged molecules compared with the zero-field case, reduced medium diffusion permeability θ for ions, as reported by the decreased D_{app}/D_{free} ratios in stronger fields (Fig. 2 *C*; Fig. S3).

Finally, we explored a similar scenario in three dimensions. For the sake of generality, we expanded the simulation arena to 10 μm and increased the diameter of diffusion-hindering spheres to the range of 0.1–1 μm (uniformly distributed; *snapshot* in Fig. 2 *D*; Fig. S4 *A*) setting time step at $\Delta t = 0.1$ μs. Similar to the cases considered above, here we found a clear, albeit less strong, reduction in the D_{app}/D_{free} ratios, hence diffusion permeability θ, either with stronger electric fields or with greater values of β (Fig. 2 *E*; Fig. S4, *B*–*F*).

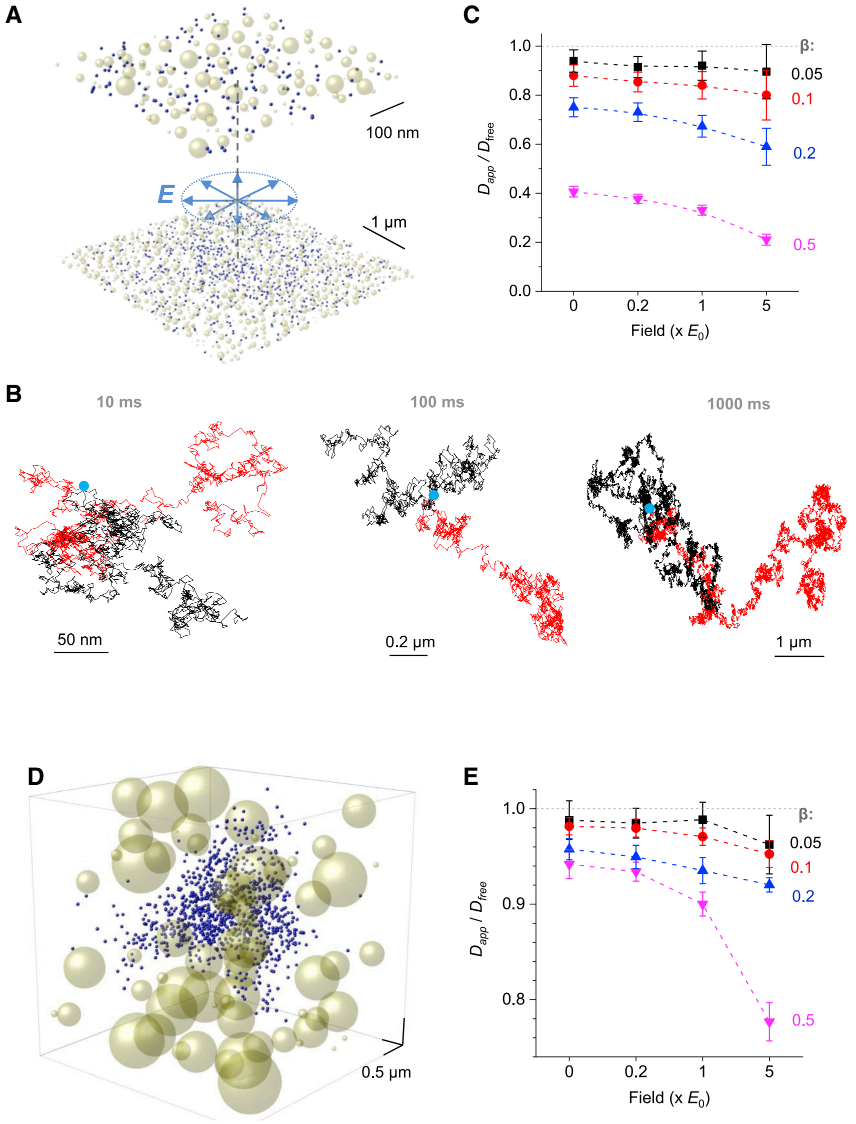

FIGURE 2 Ion-diffusion permeability of obstacle-filled media in radial electric fields. (*A*) An example of the simulated scatter of diffusing particles (*blue dots*) in a 20-nm flat cleft extending 6-μm-wide (*fragment*; two different scales as shown; the actual simulation data were rendered by OriginPro): a snapshot at 0.1 ms postrelease at the central point (*dotted line*) with $\beta = 0.2$ and $E = 0$; blue arrows indicate the radial field. (*B*) Snapshots of single-particle particle trajectories at three time points, as indicated, with $\beta = 0$ (*red*) and $\beta = 0.2$ (*black*) under no field ($E = 0$); blue dots indicate the diffusion starting point. (*C*) Statistical summary of simulations in (*A*) and (*B*) (examples of individual MC runs are shown in Fig. S3); dots indicate diffusion permeability $\theta = D_{app}/D_{free}$- (mean $\pm$ SD) values for n = 10 runs completed for each set of β- and E-values as indicated; the overall effect of either factor E (four levels) or β (four levels) on θ is at $p < 0.001$, determined by two-way ANOVA ($F_E = 45.3$, $F_\beta = 906$). (*D*) Simulation example: a scatter of diffusing particles (*blue dots*) in a 3D space (*fragment*; actual simulation data rendered by OriginPro); a snapshot at 0.1-ms postrelease at the central point (*dotted line*) with $\beta = 0.2$ of space occupied by spherical obstacles (*yellow spheres*), with electric field strength $E = 0$; radial electric field is centered at the coordinate origin. (*E*) Satistical summary of simulation experiments depicted in (*C*) (examples of individual MC runs are shown in Fig. S3); the overall effect of either factor E (four levels) or β (four levels) on θ is at $p < 0.001$, determined by two-way ANOVA ($F_E = 24.4$, $F_\beta = 31.3$); other notation is as in (*C*). To see this figure in color, go online.

We note that in these simulations, the actual dimensions are mainly for illustration purposes; the dependence between field strength and medium diffusion permeability remains unchanged when the arena geometry, diffusion coefficient, and electric field are scaled by the same time and space factor. Our data also suggest that the effect of field on medium diffusion permeability is progressively weaker under radial fields with higher dimensions, at least in the vicinity of the source, as modeled here (Fig. 3 *A*), even when the field strength is kept the same throughout the space. As mentioned above, at large distances from the source, one would expect the local field to be well approximated by the 1D case.

Geometric obstacles and classical electrodiffusion theory

It was important to assess how our MC simulation results are related to the classical NP theory. First, for the sake of generality, we tested whether positioning the field source near the diffusion source or away from it produced the same time-averaged apparent diffusion coefficient in our settings. Control MC runs confirmed that this was the case: particles showed a characteristic wave-like scatter away from the field source, showing the convergence of D_{app} for varied distance between the field and the diffusion sources (Fig. 3 *B*).

Next, we systematically compared the particle concentration profiles computed using MC simulations with those obtained using analytical solutions of the canonical NP equation (Eq. 2), which could be written in the following form:

$$\frac{1}{D}\frac{\partial C}{\partial t} = \nabla\left(\nabla C - \frac{e}{kT}C\mathbf{E}\right), \tag{3}$$

where e is elementary unit charge and k is the Boltzmann constant (Table 1). For several cases of $\beta > 0$- and E-values,

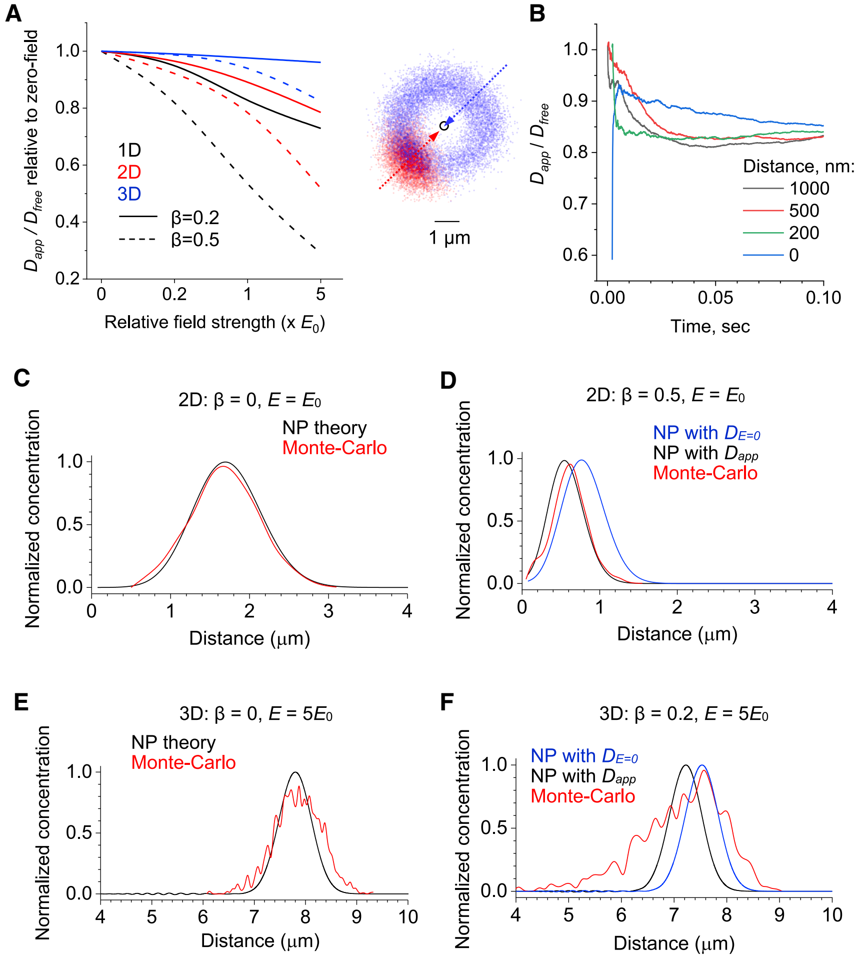

FIGURE 3 Classical electrodiffusion theory may overestimate apparent diffusivity when applied to a porous medium. (*A*) A summary of MC simulations (as in Figs. 1 and 2) showing the dependence between field strength and diffusion permeability $\theta = D_{app}/D_{free}$ (normalized to the value at $E = 0$) for two β-values in 1D uniform and 2D and 3D radial electric fields, as indicated. The strongest dependence corresponds to 1D filed at the higher β. (*B*) Image panel, an illustration of an MC test in which diffusing particles are released either at the field source (*open circle*, *blue arrow*, and *blue dots*) or at a distance of 200 nm from it (*red arrow* and *red dots*) under $E = E_0$ (10^4 V/m); a snapshot 100 μs postrelease. Graph, tests showing the time course of D_{app}/D_{free} values for particle cohorts released at different distances from the field source, as indicated, in condition as above. (*C*) The black line indicates the analytical solution of the NP equation showing a particle concentration profile (normalized to maximum) against distance to the diffusion source for 2D radial field with no obstacles ($\beta = 0$, $D = D_{free}$) under $E = E_0$ at time point $t = 0.1$ ms; the red line indicates the outcome of MC simulations in similar conditions. (*D*) Graph similar to (*C*), but with $\beta = 0.5$; blue indicates the analytical solution of the NP equation in which apparent diffusivity $D_{E=0}$ is derived by MC simulations for $E = 0$ ($\beta = 0.5$); black, a similar solution but with D_{app} calculated from MC simulations incorporating electric field $E = E_0$ (as in the graph in Fig. 2 *C*, for $E = 1 \times E_0$, $\beta = 0.5$); red indicates the outcome of MC simulations, as indicated. (*E*) Graph similar to (*C*) but for 3D radial field with $\beta = 0.2$ and $E = 5E_0$, as indicated. Other notations are as in (*C*). (*F*) Graph similar to (*D*) but for 3D radial field with $\beta = 0.2$ and $E = 5E_0$. Other notations are as in (*D*); blue indicates the analytical solution of the NP equation in which $D_{E=0}$ is derived by MC simulations for $E = 0$ ($\beta = 0.2$); black indicates a similar solution but with D_{app} derived by MC simulations under $E = 5E_0$ (as in Fig. 2 *E*, data point at $E = 1 \times 5E_0$, $\beta = 0.2$). To see this figure in color, go online.

we thus calculated an analytical solution of the NP equation at a certain time point ($t = 100$ μs), first for diffusivity $D_{E=0}$ estimated by MC simulations under $E = 0$. This solution was compared with the analytical solution calculated for diffusivity D_{app} estimated from MC simulations under the corresponding $E > 0$-value. These cases were also compared with the concentration profile obtained directly by MC simulations under the same conditions.

As expected, in all cases under $E > 0$, the concentration profiles showed a characteristic wave that was spreading away from the diffusion source (Fig. 3, *C–F*). Reassuringly, in a free medium ($\beta = 0$), MC simulations provided an excellent match with the analytical NP solution for a given *E* (Fig. 3, *C* and *E*). In contrast, in an obstacle-filled medium ($\beta > 0$), the classical NP theory overestimated the effective diffusivity values, with the discrepancy increasing with stronger fields (Fig. 3, *D* and *F*). In other words, the outcome of MC simulations could be satisfactorily described using the analytical NP theory but with a diffusion coefficient corrected for the field-dependent transfer retardation.

Physical testing of electrodiffusion in a porous medium

To test our theoretical prediction that the ion conductance of porous media is field dependent, we designed and implemented a simple physical experiment. We measured electrolyte currents between the opposite sides (flat parallel walls serving as electrodes) of a narrow rectangular chamber filled with microscopic glass spheres (Fig. 4, *A* and *B*; Materials and methods), a design somewhat similar to that explored previously (36). The space fraction β occupied by the spheres was evaluated by monitoring electrolyte displacement in the chamber upon sphere filling, $\beta = 0.30 \pm 0.02$. We used 10-ms square voltage pulses over a range of voltages that would generate inside the chamber electric fields compatible with those in the brain tissue (Fig. 4 *C*; Materials and methods). The three solutions tested were water, NaCl (153 mM), and standard ACSF (Materials and methods).

As expected, these measurements showed monotonic, quasilinear dependencies between voltage (electric field) and

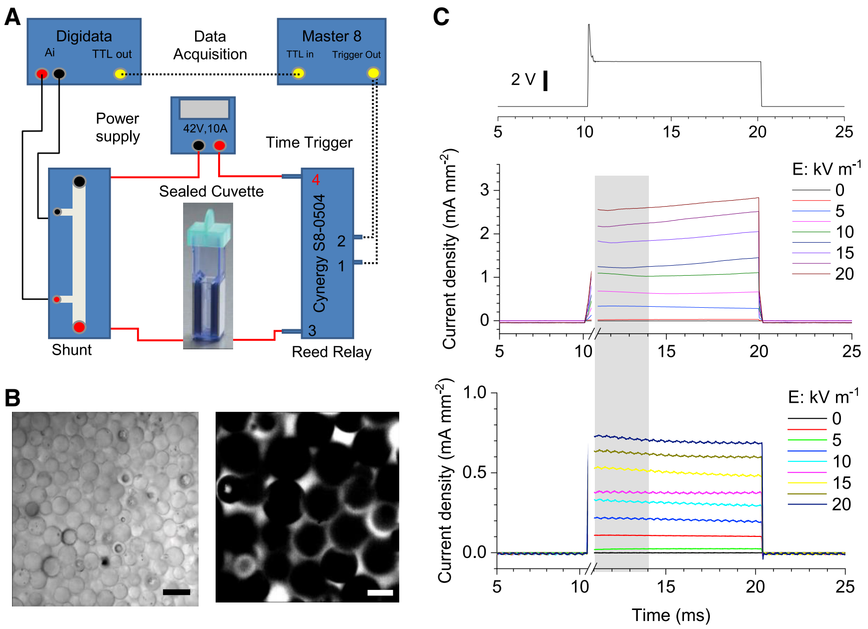

FIGURE 4 Experimental testing of obstacle-filled medium conductivity under varied electric fields. (*A*) An experiment schematic. The test solution, with or without glass beads, is placed in a cuvette equipped with flat side electrodes (Materials and methods). Controlled application of ΔV voltage steps to the electrodes induces electric current measured digitally, as depicted. (*B*) Electrolyte solution (NaCl) filled with densely packed microscopic glass beads is shown in transmitted light (800 nm) at the top layer (*left*; scale bar, 50 μm) and as a fluorescent image (*right*; solution containing Alexa Fluor 488, two-photon excitation 800 nm; scale bar, 20 μm). (*C*) The time course of voltage pulse application (an example; *top*) and the current response in a fee-medium (*middle*) and sphere-filled NaCl solution (*bottom*). Gray shading indicates the sampled response area (first 3 ms after the pulse artifact). To see this figure in color, go online.

electrolyte current in either free-space or sphere-filled chambers (Fig. 5 *A*; pure water provided control measurements). However, because physical properties of electrolytes could be affected by electric field, it was important to compare these measurements directly between free and obstacle-filled cases under the same field strength: the ratio between the corresponding current density values (porous-to-free) should represent $\theta = D_{app}/D_{free}$. We thus found that θ decreased with greater strengths of the electric field, both for ACSF and NaCl solutions (Fig. 5 *B*, *data points*). This dependence was similar to that obtained in MC simulations of 1D electrodiffusion for β-values roughly between 0.3 and 0.4 (Fig. 5 *B*, *dotted lines*), which was a remarkable correspondence given that the volume fraction occupied by glass spheres in these experiments was $\beta \sim 0.3$. The slight shift of theoretical curves toward higher values of β (Fig. 5 *B*) was likely because simulated spheres were allowed to overlap, thus leading to somewhat higher space tortuosity. It appeared therefore that the electrical resistance of tested electrolytes in a porous space depended on the external electric field, in good correspondence with our theoretical predictions.

Theoretical insight

Given the unlimited variety of porous-medium geometries, it would not seem feasible to generalize our findings as a fully-fledged electrodiffusion theory. However, we sought to explore the first principles by considering geometric obstacles as barriers in the field-potential profile that drives ion-diffusion transfer. For 1D diffusion (which is 3D diffusion in a long narrow cylinder), the mean-square displacement of a free Brownian particle increases linearly with time t, $\langle x^2 \rangle \sim t$, plus an additional drift when the electric field is present (Fig. 5 *C*, *i* and ii; *red lines* illustrate potential profiles for stronger and weaker field). When the diffusing particle encounters an obstacle or a trap, it takes additional time τ to escape it so that particle displacement over time t is reduced to $\langle x^2 \rangle \sim t^{\alpha(\tau)}$, where $0 < \alpha(\tau) < 1$. The obstacle-imposed additional dwell time τ depends on the characteristic height of the potential barrier (or trap) H (Fig. 5 *C*, *iii*). Generally, for diffusion under an electric field (electrodiffusion), H scales with field strength E (Fig. 5 *C*, iii; *red lines* illustrate how the potential barrier scales with the field strength). It has long been established that 1D stochastic diffusion in field E can be described by the canonical Dynkin's operator $\Psi = \frac{qDFE}{RT}\frac{d}{dx} + D\frac{d^2}{dx^2}$ (p. 241 in (37)), in which the first and second terms define electric drift and free diffusion, respectively; R is the gas constant, T is the temperature, and F is the Faraday constant. When field E is directed against the particle's escape over a certain barrier, the mean exit (dwell) time τ is given by the boundary value problem $-\psi\tau = 1$ with the Dirichlet boundary condition. This leads to the following steady-state equation (38):

$$D\left(\frac{\partial^2 \tau}{\partial x^2} + \frac{qFE}{RT}\frac{\partial \tau}{\partial x}\right) = -1, \tag{4}$$

with the boundary conditions

$$\tau(x = 0) = 0, \; \frac{\partial \tau}{\partial x}(x = H) = 0,$$

where x in this case denotes the initial particle's coordinate (between 0 and H). Its solution for τ is

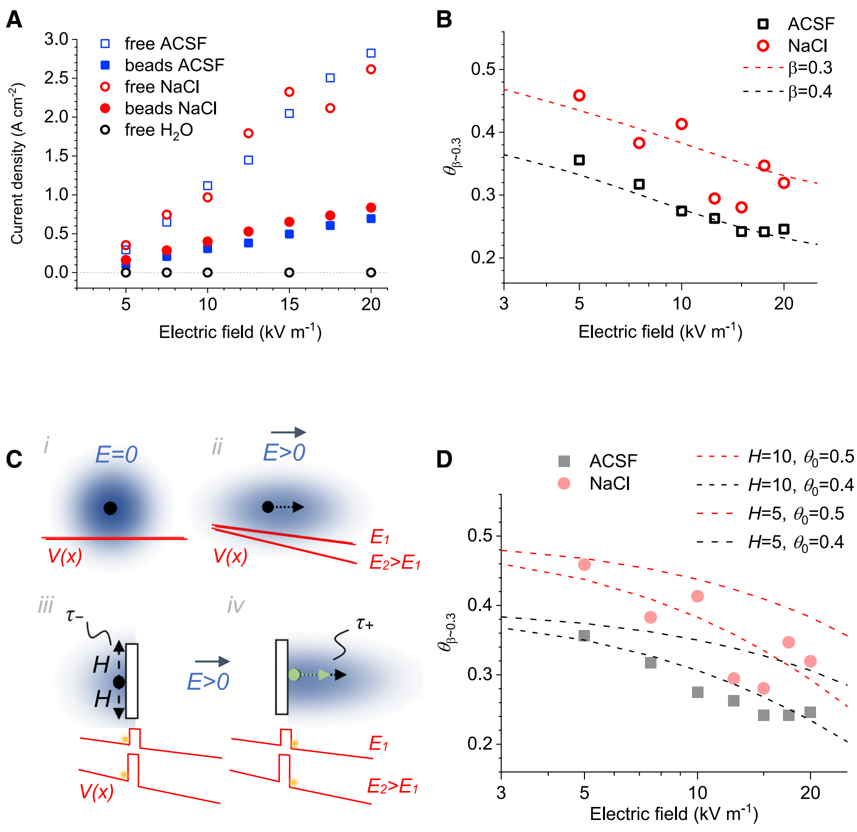

FIGURE 5 Electric conductivity of porous tissue decreases with stronger electric fields. (*A*) The recorded current density plotted against electric field in ACSF and NaCl solutions, with and without densely packed microscopic glass beads (as in Fig. 4), as indicated; control recordings with deionized water. (*B*) Relative porous-medium conductivity (ratio between porous- and free-medium conductivity values) for ACSF and NaCl electrolytes is plotted against the electric field. Dotted lines indicate theoretical dependencies calculated for ions diffusing in a 1D uniform electric field (as in Fig. 1) for $\beta = 0.4$ and $\beta = 0.3$ (experimental value in Fig. 4 tests), as indicated. (*C*) A schematic illustrating (not to scale) a theoretical approach (Eqs. 4, 5, 6, 7, and 8); blue shading indicates the probability density for particle occurrence (*dot*, starting point) under no electric field (*i*), under uniform field E (*ii*; *dotted arrow*, average drift), facing the barrier of radius H (*iii*; $\tau-$, maximal dwell time "lost" to navigate the barrier), and behind the barrier (*iv*; *green dotted arrow*, no-obstacle particle drift, as in *ii*; *black arrow*, average drift increased because of the "gained" time τ_+); red lines illustrate profiles of field-potential $V(x)$ in 1D space for weaker (E_1) and stronger (E_2) fields, with orange dots representing particles facing (*iii*) or past (*iv*) the potential barrier. (*D*) Experimental data are given as in (*B*) but with the theoretical dependencies (*dashed* and *dotted-dashed lines*) calculated from the analytical solution (Eq. 8) for several values of H and θ_0, as indicated. To see this figure in color, go online.

$$\tau(x) = \frac{e^{(-\kappa EH)}\left(1 - e^{\kappa Ex} + e^{\kappa EH}\kappa Ex\right)}{(\kappa E)^2 D}, \tag{5}$$

where $\kappa = RT/F$. For $x = H$ (displacement to the edge of the barrier H), Eq. 5 becomes

$$\tau_-(H) = \frac{\kappa EH - 1 + e^{(-\kappa EH)}}{(\kappa E)^2 D}. \tag{6}$$

Equation 6 thus describes the average barrier-imposed dwell time τ_- (Fig. 5 *C*, *iii*). This time defines diffusion deceleration (time delay) when a particle faces an obstacle so that field E hinders its diffusion escape. In this case, particle displacement is reduced compared with free diffusion. On the same space-time scale, when the particle is behind the trap in the direction of E or otherwise if the field is reversed (to $-E$), it gains an additional drift along E because any stochastic movement in the opposite direction is prevented by the barrier (Fig. 5 *C*, *iv*). This increase in drift, when compared with free-medium diffusion, translates into time gain:

$$\tau_+(H) = \frac{-\kappa EH - 1 + e^{(\kappa EH)}}{(\kappa E)^2 D}. \tag{7}$$

Then, the relative medium conductivity estimate θ under field E could be estimated by the expression

$$\theta(H) = \theta_0 \frac{\tau_-(H)}{\tau_+(H)} = \theta_0 \frac{\kappa EH - 1 + e^{(-\kappa EH)}}{-\kappa EH - 1 + e^{(\kappa EH)}}, \tag{8}$$

where θ_0 is the relative medium conductance (porous versus obstacle-free) under zero field ($E = 0$). In our experimental settings (Fig. 4), the θ_0 value is estimated between 0.4 and 0.5 (Fig. 5 *B*) so that Eq. 8 has one free parameter H. It turns out that with the H-values set between 5 and 10 μm, Eq. 8 provides a reasonably close prediction of the experimental dependence between relative conductivity θ and field strength (Fig. 5 *D*).

What could be the meaning of H-values in our experimental test (Fig. 4)? Several studies suggest that the space tortuosity for a sphere-packed medium with $\beta = 0.3$–0.4 ranges between 1.3 and 1.5 (39–41). This implies a 30–50% increase in the particle's diffusing path compared with an obstacle-free medium. With the characteristic sphere radius in our tests of ~15 μm (Fig. 4 *C*), this increase corresponds to ~5–7 μm of additional path per obstacle, which falls within the range of "best-fit" H-values of 5 and 10 μm. Whether Eq. 8 and the meaning of H hold in

more general cases remains an open and intriguing question.

DISCUSSION

The main finding of this study is that diffusion permeability of porous (obstacle-filled) media for charged particles could depend on the macroscopic electric field. MC simulations predicted that the apparent diffusion coefficient, or electrical conductivity, in such media decreases with stronger electric fields that otherwise accelerate diffusion transfer. This dependence is enhanced with increased geometric hindrance or decreased porosity. Our data also suggest that the additional diffusion hindrance due to the electric field effect is weaker in radial compared with uniform field (at least when close to the field source), even when the field strength remains the same throughout the space. The latter observation might help explain conclusions of an earlier study (42) in which geometric considerations suggested a longer diffusion path for "1D diffusion" compared with point-source diffusion in 2D or 3D; in fact, these earlier conclusions should have referred to ion diffusion in a strong uniform (1D) field compared with radial fields. One could also consider a limiting case when the electric field drift dwarfs any stochastic Brownian effects; in that case, space dead-ends in the porous media (4,43,44) could entirely prevent particle transfer.

Modeling electrodiffusion

The MC algorithms for diffusion and electrodiffusion employed here have previously been tested and validated systematically against experimental recordings, including submillisecond fast-exchange receptor probing in outside-out and nucleated (whole-cell) membrane patches (25,26). In this study, we compared the MC simulation outcome with the analytical solutions of the NP equation in several key settings and found good correspondence. Throughout our analyses, we assumed no interactions between diffusing particles and the spherical obstacles, such as any electrostatic or electroosmotic influence; we tried to focus on the case of small ions diffusing, on the microscopic scale, in the weak (fully dissociated) electrolyte filled with macromolecules and other inert nanoscopic obstacles. This may not necessarily be the case when particles diffuse in sufficiently narrow clefts between charged surfaces, which may give rise to further interference with their transfer (20,22). In this context, our MC algorithms enabled diffusing particles to navigate individual obstacles, roughly in correspondence with the distortions of electric field near the surface of dielectric (low permittivity) spheres. Although it would be important to have a more rigorous assessment of particle behavior in the vicinity of obstacles, the particle concentration profiles generated by our MC simulations appeared in good agreement with the NP solutions incorporating the apparent diffusion coefficient.

We tested our theoretical predictions using a simple physical experiment in which the relationship between electric current and external field was measured in controlled conditions involving glass-sphere-filled electrolyte solutions. The electrolyte strength was sufficiently low (much below 0.2 M) to assume free ion mobility (full dissociation) without ion-ion interactions, whereas the porosity value was high enough ($\sim$0.7) to ignore electrostatic interactions with the sphere walls. The shape and the material of obstacles can also affect interpretation of the results. In our tests, soda glass microbeads were selected for the several reasons. First, we aimed to match the geometry (if not size) of obstacles in our MC simulations. Second, solid glass material has a high crush strength, which makes it suitable for high-density packing. Third, soda glass helped to avoid polarization effects in the spheres if compared with other materials like polystyrene or coated metals. Although the experiment did not replicate the dimensions of the MC simulation settings, we considered it suitable enough to address the underlying principle. In addition, our control MC simulations suggested that having either overlapping spheres (as we did throughout our simulation tests) or nonoverlapping spheres (in the experiment) had indistinguishably similar effects on the apparent diffusion coefficient, at least for $\beta < 0.5$.

Potential implications for electrophysiology

Across the areas of biology (and other sciences), porous media come with highly variable geometries and electrodiffusion scenarios. This makes it difficult to suggest a generalized theory, although significant progress has been made in the description of ion flows in media consisting of tightly packed obstacles separated by narrow pores (such as porous rocks, sediments, ceramics, etc.) (20,21). Recently, an elegant study has introduced a Kirchhoff-NP formulism to model macroscopic electrodiffusion in the interstitial space surrounding nerve cells (22). On the nanoscale in the brain, a better understanding of electrodiffusion phenomena is beginning to emerge (23,45), reflecting a present gap in our knowledge (2,46). In this context, much less attention has been paid to the potential influence of electric fields on the conductive properties of obstacle-filled electrolytes in biological tissues. The potential importance of this issue stems from the clear dependence between extracellular ion current (both diffusive and resistive components) and the external field frequency in brain tissue (15,17). Such nonohmic properties of the brain extracellular medium can be related to the local structural inhomogeneities so that the timescale of changes in the field strength and/or direction affects local ion flow differently depending on the size of local obstacles (16). This appears consistent, in principle, with our conclusions that relate field strength (and β) to medium diffusion permeability.

One potentially important consequence of having local nonohmic properties of the brain tissue is the quantitative interpretation of electrocorticography of field-potential recordings. Such recordings reflect the strength, spatial distribution, and preferred orientation of local current sources and sinks inside the tissue volume conductor; estimating these parameters from the (multipoint) field-potential readout constitutes the classical inverse problem of brain electrophysiology (18). Introducing nonohmic tissue properties that might arise from our findings might therefore lead to somewhat differing estimates pertaining to the arrangement and strength of active current sources across the frequency spectrum (16,18). This could in turn affect our understanding of electrical neural network activity based on the current-source reconstruction from electrophysiological recordings.

Here, we have attempted a first-principle theoretical treatment in which geometrical obstacles are considered potential barriers in the homogenous electric field. It appears that under plausible assumptions and within the tested range of experimental parameters, the theory provides a reasonable prediction of the dependence between electric field strength and relative reduction in conductivity observed experimentally. Clearly, further tests should answer the question how general these theoretical findings are.

SUPPORTING MATERIAL

Supporting material can be found online at https://doi.org/10.1016/j.bpj.2021.02.012.

AUTHORS CONTRIBUTIONS

L.P.S. designed and carried out the MC simulations and developed the analytical theory. K.Z. designed and implemented the physical tests. L.P.S., K.Z., and D.A.R. analyzed the data. D.A.R. narrated the study, designed selected approaches, and wrote the manuscript, which was contributed to by all authors.

ACKNOWLEDGMENTS

Optimization and parallelization of computing algorithms were implemented by AMC Bridge LLC (Waltham, MA).

This work was supported by the Wellcome Trust Principal Fellowship Award (212251_Z_18_Z), European Research Council Advanced Grant (323113), and European Commission NEUROTWIN grant (857562) to D.A.R.

REFERENCES

1. Syková, E., and C. Nicholson. 2008. Diffusion in brain extracellular space. *Physiol. Rev.* 88:1277–1340.
2. Savtchenko, L. P., M. M. Poo, and D. A. Rusakov. 2017. Electrodiffusion phenomena in neuroscience: a neglected companion. *Nat. Rev. Neurosci.* 18:598–612.
3. Nicholson, C., and L. Tao. 1993. Hindered diffusion of high molecular weight compounds in brain extracellular microenvironment measured with integrative optical imaging. *Biophys. J.* 65:2277–2290.
4. Hrabe, J., S. Hrabetová, and K. Segeth. 2004. A model of effective diffusion and tortuosity in the extracellular space of the brain. *Biophys. J.* 87:1606–1617.
5. Zheng, K., A. Scimemi, and D. A. Rusakov. 2008. Receptor actions of synaptically released glutamate: the role of transporters on the scale from nanometers to microns. *Biophys. J.* 95:4584–4596.
6. Zhang, H., and A. S. Verkman. 2010. Microfiberoptic measurement of extracellular space volume in brain and tumor slices based on fluorescent dye partitioning. *Biophys. J.* 99:1284–1291.
7. Arizono, M., V. V. G. K. Inavalli, …, U. V. Nägerl. 2020. Structural basis of astrocytic Ca^{2+} signals at tripartite synapses. *Nat. Commun.* 11:1906.
8. Tønnesen, J., V. V. G. K. Inavalli, and U. V. Nägerl. 2018. Super-resolution imaging of the extracellular space in living brain tissue. *Cell.* 172:1108–1121.e15.
9. Zheng, K., T. P. Jensen, …, D. A. Rusakov. 2017. Nanoscale diffusion in the synaptic cleft and beyond measured with time-resolved fluorescence anisotropy imaging. *Sci. Rep.* 7:42022.
10. Cai, J. C., W. Wei, …, D. A. Wood. 2017. Electrical conductivity models in saturated porous media: a review. *Earth Sci. Rev.* 171:419–433.
11. Bussian, A. E. 1983. Electrical conductance in a porous-medium. *Geophysics.* 48:1258–1268.
12. Bédard, C., and A. Destexhe. 2009. Macroscopic models of local field potentials and the apparent 1/f noise in brain activity. *Biophys. J.* 96:2589–2603.
13. Li, G., and B. M. Fu. 2011. An electrodiffusion model for the blood-brain barrier permeability to charged molecules. *J. Biomech. Eng.* 133:021002.
14. Bédard, C., and A. Destexhe. 2011. Generalized theory for current-source-density analysis in brain tissue. *Phys. Rev. E Stat. Nonlin. Soft Matter Phys.* 84:041909.
15. Gomes, J. M., C. Bédard, …, A. Destexhe. 2016. Intracellular impedance measurements reveal non-ohmic properties of the extracellular medium around neurons. *Biophys. J.* 110:234–246.
16. Nelson, M. J., C. Bosch, …, P. Pouget. 2013. Microscale inhomogeneity of brain tissue distorts electrical signal propagation. *J. Neurosci.* 33:2821–2827.
17. Logothetis, N. K., C. Kayser, and A. Oeltermann. 2007. In vivo measurement of cortical impedance spectrum in monkeys: implications for signal propagation. *Neuron.* 55:809–823.
18. Buzsáki, G., C. A. Anastassiou, and C. Koch. 2012. The origin of extracellular fields and currents–EEG, ECoG, LFP and spikes. *Nat. Rev. Neurosci.* 13:407–420.
19. Zhang, Y. Y., G. S. Wang, …, X. L. Dong. 2008. The model of electric field dependent dielectric properties for porous ceramics. *J. Appl. Phys.* 103:114103.
20. Di Fraia, S., N. Massarotti, and P. Nithiarasu. 2018. Modelling electro-osmotic flow in porous media: a review. *Int J Numer Method H.* 28:472–497.
21. Szyszkiewicz, K., J. J. Jasielec, …, R. Filipek. 2017. Modeling of electrodiffusion processes from nano to macro scale. *J. Electrochem. Soc.* 164:E3559–E3568.
22. Solbrå, A., A. W. Bergersen, …, G. Halnes. 2018. A Kirchhoff-Nernst-Planck framework for modeling large scale extracellular electrodiffusion surrounding morphologically detailed neurons. *PLoS Comput. Biol.* 14:e1006510.
23. Holcman, D., N. Hoze, and Z. Schuss. 2011. Narrow escape through a funnel and effective diffusion on a crowded membrane. *Phys. Rev. E Stat. Nonlin. Soft Matter Phys.* 84:021906.
24. Biess, A., E. Korkotian, and D. Holcman. 2011. Barriers to diffusion in dendrites and estimation of calcium spread following synaptic inputs. *PLoS Comput. Biol.* 7:e1002182.

25. Savtchenko, L. P., S. Sylantyev, and D. A. Rusakov. 2013. Central synapses release a resource-efficient amount of glutamate. *Nat. Neurosci.* 16:10–12.

26. Sylantyev, S., L. P. Savtchenko, …, D. A. Rusakov. 2013. Spike-driven glutamate electrodiffusion triggers synaptic potentiation via a homer-dependent mGluR-NMDAR link. *Neuron.* 77:528–541.

27. Savtchenko, L. P., S. N. Antropov, and S. M. Korogod. 2000. Effect of voltage drop within the synaptic cleft on the current and voltage generated at a single synapse. *Biophys. J.* 78:1119–1125.

28. Savtchenko, L. P., and D. A. Rusakov. 2007. The optimal height of the synaptic cleft. *Proc. Natl. Acad. Sci. USA.* 104:1823–1828.

29. Conway, J. H., and N. J. A. Sloane. 1995. What are all the best sphere packings in low dimensions. *Discrete Comput. Geom.* 13:383–403.

30. Kimberly, E. E. 1939. Electrical Engineering. The Haddon Craftsmen Inc, Scranton, PA.

31. Levitt, D. G. 1985. Strong electrolyte continuum theory solution for equilibrium profiles, diffusion limitation, and conductance in charged ion channels. *Biophys. J.* 48:19–31.

32. Holcman, D., and Z. Schuss. 2017. 100 years after Smoluchowski: stochastic processes in cell biology. *J. Phys. A Math. Theor.* 50:093002.

33. Hrabetová, S. 2005. Extracellular diffusion is fast and isotropic in the stratum radiatum of hippocampal CA1 region in rat brain slices. *Hippocampus.* 15:441–450.

34. Tao, L., and C. Nicholson. 2004. Maximum geometrical hindrance to diffusion in brain extracellular space surrounding uniformly spaced convex cells. *J. Theor. Biol.* 229:59–68.

35. Weissberg, H. L. 1963. Effective diffusion coefficient in porous media. *J. Appl. Phys.* 34:2636–2639.

36. Eberhardt, W., D. Woidich, and A. Reichenbach. 1990. Determination of the extracellular tortuosity in nuclear layers of the central nervous system by resistance measurements on a geometrical model. *J. Hirnforsch.* 31:1–11.

37. Dynkin, E. B. 1965. Characteristic operators of Markov processes differential generators of diffusion processes. *In* Markov Processes. Die Grundlehren der Mathematischen Wissenschaften, *Volume 12*. E. B. Dynkin, ed.. Academic Press, p. 133.

38. Pavliotis, G. A. 2014. Stochastic Processes and Applications: Diffusion Processes, the Fokker-Planck and Langevin Equations. Texts in Applied MathematicsVolume 60. Springer, New York.

39. Ahmadi, M. M., S. Mohammadi, and A. N. Hayati. 2011. Analytical derivation of tortuosity and permeability of monosized spheres: a volume averaging approach. *Phys. Rev. E Stat. Nonlin. Soft Matter Phys.* 83:026312.

40. Boutin, C., and C. Geindreau. 2010. Periodic homogenization and consistent estimates of transport parameters through sphere and polyhedron packings in the whole porosity range. *Phys. Rev. E Stat. Nonlin. Soft Matter Phys.* 82:036313.

41. Pisani, L. 2011. Simple expression for the tortuosity of porous media. *Transp. Porous Media.* 88:193–203.

42. Rusakov, D. A., and D. M. Kullmann. 1998. Geometric and viscous components of the tortuosity of the extracellular space in the brain. *Proc. Natl. Acad. Sci. USA.* 95:8975–8980.

43. Hrabetová, S., J. Hrabe, and C. Nicholson. 2003. Dead-space microdomains hinder extracellular diffusion in rat neocortex during ischemia. *J. Neurosci.* 23:8351–8359.

44. Binder, D. K., M. C. Papadopoulos, …, A. S. Verkman. 2004. In vivo measurement of brain extracellular space diffusion by cortical surface photobleaching. *J. Neurosci.* 24:8049–8056.

45. Cartailler, J., and D. Holcman. 2018. Electrical transient laws in neuronal microdomains based on electro-diffusion. *Phys. Chem. Chem. Phys.* 20:21062–21067.

46. Holcman, D., and R. Yuste. 2015. The new nanophysiology: regulation of ionic flow in neuronal subcompartments. *Nat. Rev. Neurosci.* 16:685–692.

Biophysical Journal, Volume *120*

Supplemental information

Conductance of porous media depends on external electric fields

Leonid P. Savtchenko, Kaiyu Zheng, and Dmitri A. Rusakov

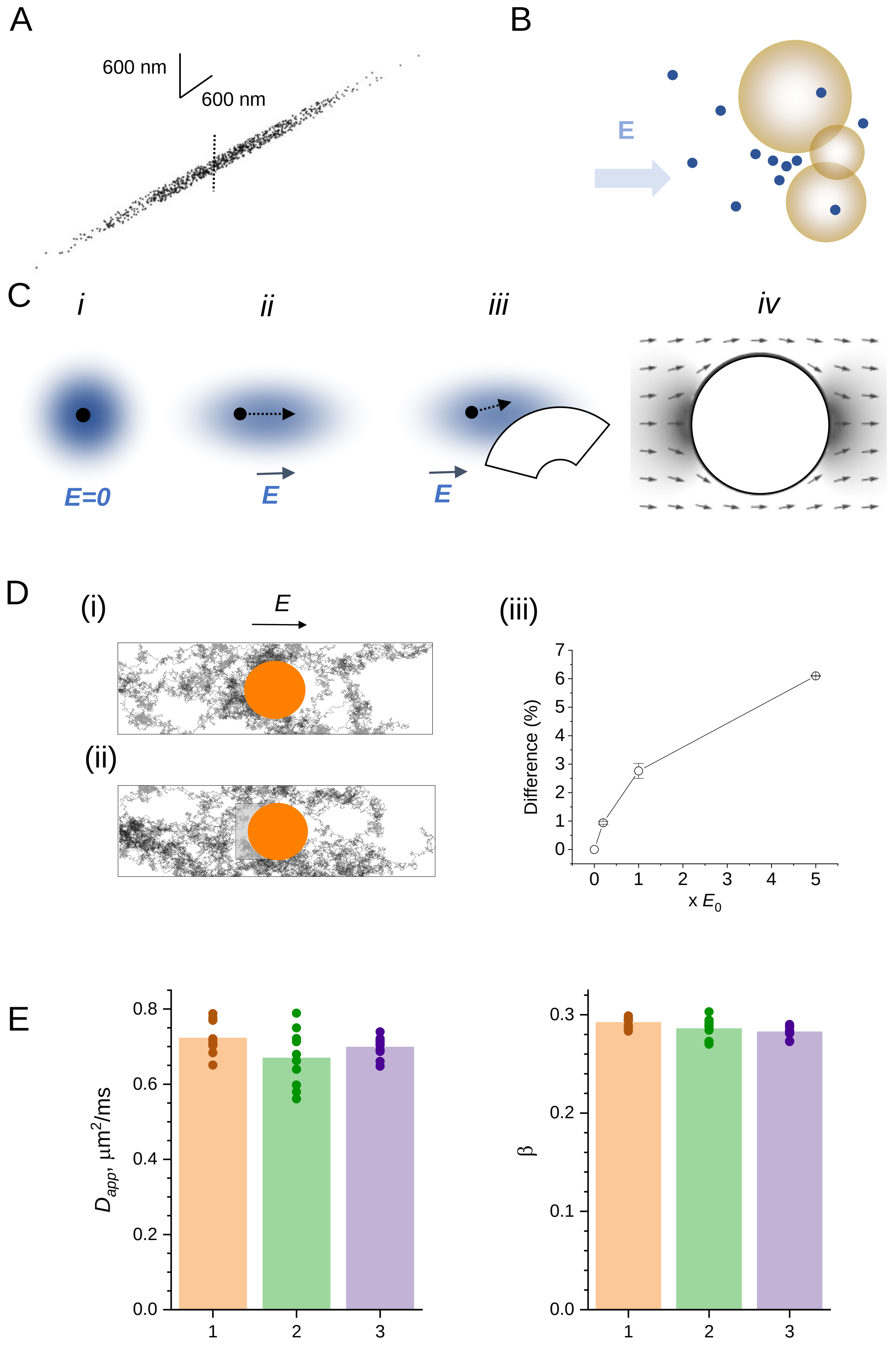
A
600 nm
600 nm
B
E
C
i
ii
iii
iv
E=0
E
E
D
(i)
E
(ii)
(iii)
Difference (%)
x E_0
E
D_{app}, µm²/ms
β

Supplementary Figure S1. MC simulations of particle diffusion under uniform electric filed.

(A) Simulation example: snapshot of the scatter of diffusing particles (dots) along a 0.1 µm wide - 10 µm long cylinder, 0.1 ms post-release from the centroid (dotted line); space fraction occupied by spherical obstacles (3-33 nm diameter, not shown) is β = 0.2, no electric field (*E* = 0); actual simulation data rendered by OriginPro.

(B) A schematic illustrating the possibility of 'dead-ends' occurring in the scatter of overlapping spheres (orange circles) that might trap molecules (blue dots) diffusing under field $E > 0$ (blue arrow).

(C) Schematic illustrating (not to scale), average behaviour of a Brownian particle; blue shade, probability density of particle occurrence (dot, starting point), under no electric field (*i*), under uniform field *E* (*ii*; dotted arrow, average drift), and facing a spherical obstacle barrier (*iii*); the latter scenario is similar to the local direction of electric field (*iv*; small arrows, standard example of field deviation near a quasi-dielectric sphere under uniform external field in a high-permittivity medium; grey shadow, relative field strength; (https://demonstrations.wolfram.com/DielectricSphereInAUniformElectricField/,polarisation effects are ignored), or otherwise the directional vector field of a hydrodynamic; flow near a round obstacle.

(D) MC simulation test ('flat' 1D diffusion) illustrating that electric field distortion near a circular obstacle (i.e., partial shielding from field) has little impact on the calculated diffusivity under a homogenous electric field: (*i*) trajectory snapshot of particle diffusion (n = 10 particles / trials, starting point at the left-boundary midpoint) around a circular obstacle, under field $E = 5E_0$; (*ii*) Example as in (*i*), but with a field-shielding area (grey shade) extending to ~1.3 of the obstacle radius, where $E = 0$: in a simple case of dielectric spheres in a uniform electric field, the field configuration at >1.3 radius from the sphere midpoint approaches the uniform field away from the sphere, across a wide range of dielectric constants (e.g., https://demonstrations.wolfram.com /DielectricSphereInAUniformElectricField/); (*iii*) Summary, the percentage difference (mean ± SEM, n = 10) between D_{app} values calculated for cases shown in (*i*) and (*ii*), for varied values of *E*. Key model parameters: circle radius 30 nm, 'flat tube' width 100 nm, $E_0 = 10^4$ V/m, $D_{free} = 1$ µm^2 ms^{-1}.

(E) MC simulation test, illustrating estimation of the average diffusivity (left graph, 1D case, n = 10 simulation runs) for three different arrangement of spherical obstacles under similar β ~ 0.29 (mean ± SD): (1) 8000 non-overlapping spheres, radius 10 nm ($D_{app} = 0.722 \pm 0.043$ µm^2/ms); (2) overlapping spheres with the radius distributed evenly between 3-30 nm ($D_{app} = 0.669 \pm 0.076$ µm^2/ms); and (3) 3500 non-overlapping spheres, radius of 14.2 nm ($D_{app} = 0.698 \pm 0.028$ µm^2/ms); dots, individual MC simulation runs. MC simulations for $\beta \sim 0.4$ gave $D_{app} = 0.55 \pm 0.13$ µm^2/ms and 0.51 ± 0.13 (mean ± SD; n = 10 runs) for cases (1) and (2), respectively.

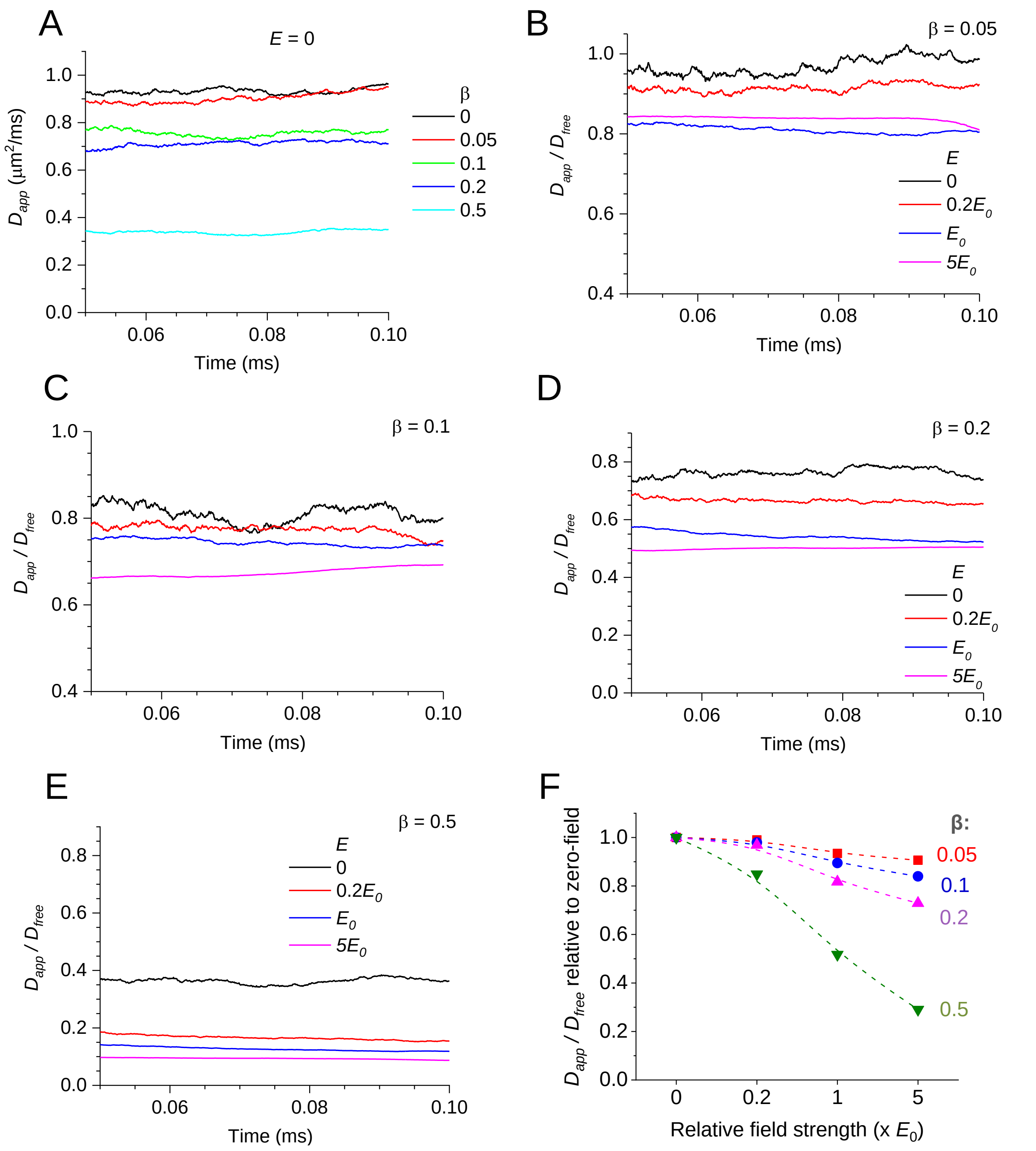

Supplementary Figure S2. MC simulations estimating apparent permeability of obstacle-filled medium in 1D electric field.

(A) Example of a MC simulation run: time course of apparent diffusivity D_{app} (along the cylinder as) under E = 0 (repeat of the test shown in Fig. 1B).

(B-E) Examples of MC simulation runs: time course of medium permeability $\theta = D_{app} / D_{free}$ (narrow cylinder), estimated for different values of β and E as indicated ($E_0 = 10^4$ V/m).

(F) A summary of MC simulations showing the dependence between field strength and permeability θ (normalised to the value at E = 0), under 1D uniform electric field, for several β values, as indicated**.**

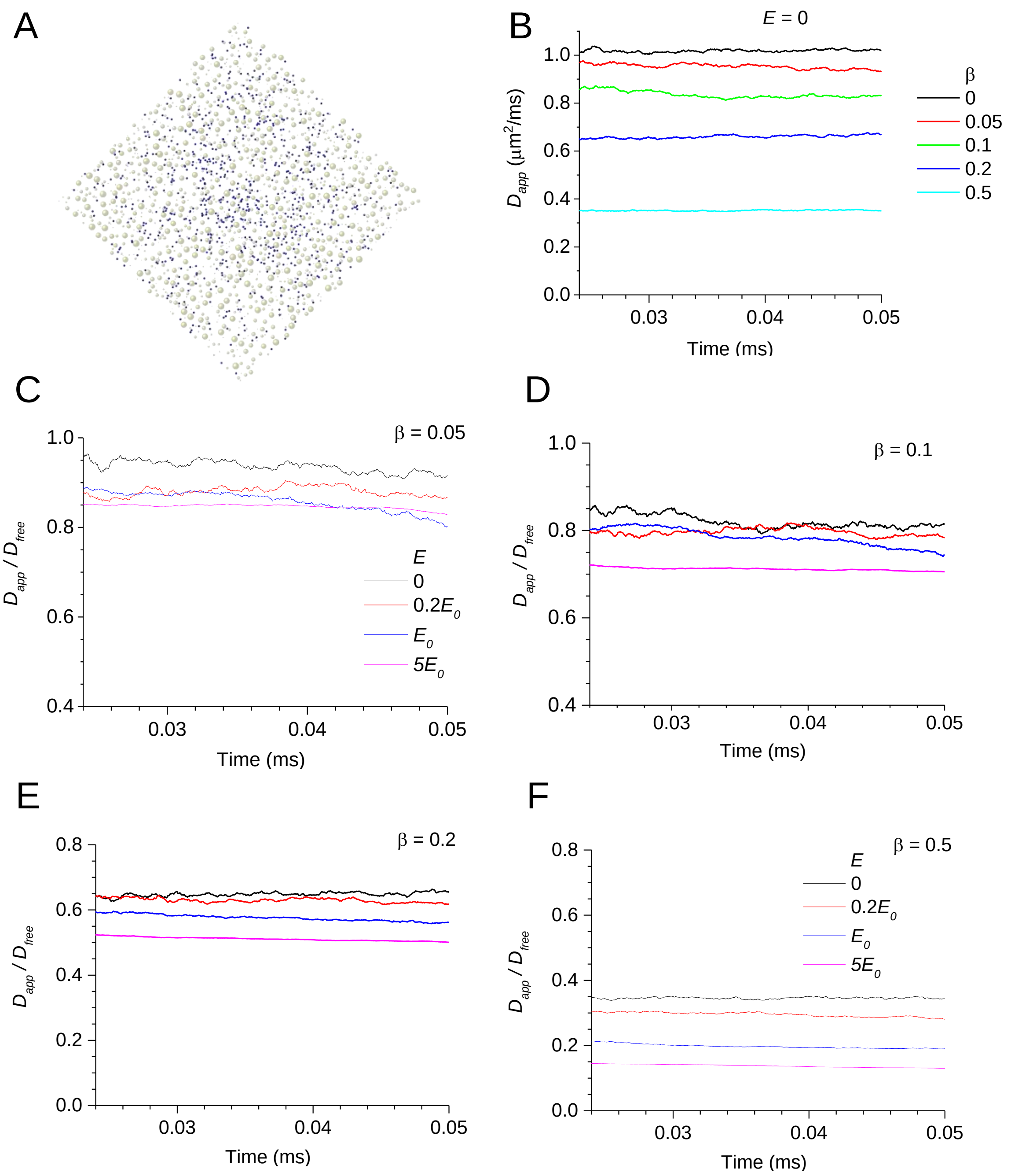

Supplementary Figure S3. Estimation of medium permeability for ions in a flat obstacle-filled cleft under a 2D radial field.

(A) Simulation example: snapshot of the scatter of diffusing particles (dots) in a flat 20 nm wide cleft along, 0.1 ms post-release from the centre; as in Fig. 2A (4 x 4 μm arena), shown as a 2D projection, for clarify. Other notations are as in Fig. 2A.

(B) Example of a MC simulation run: time course of apparent diffusivity D_{app} in the flat cleft under $E = 0$.

(C-F) Examples of MC simulation runs: time course of medium permeability $\theta = D_{app} / D_{free}$, estimated for different values of β and E as indicated ($E_0 = 10^4$ V/m).

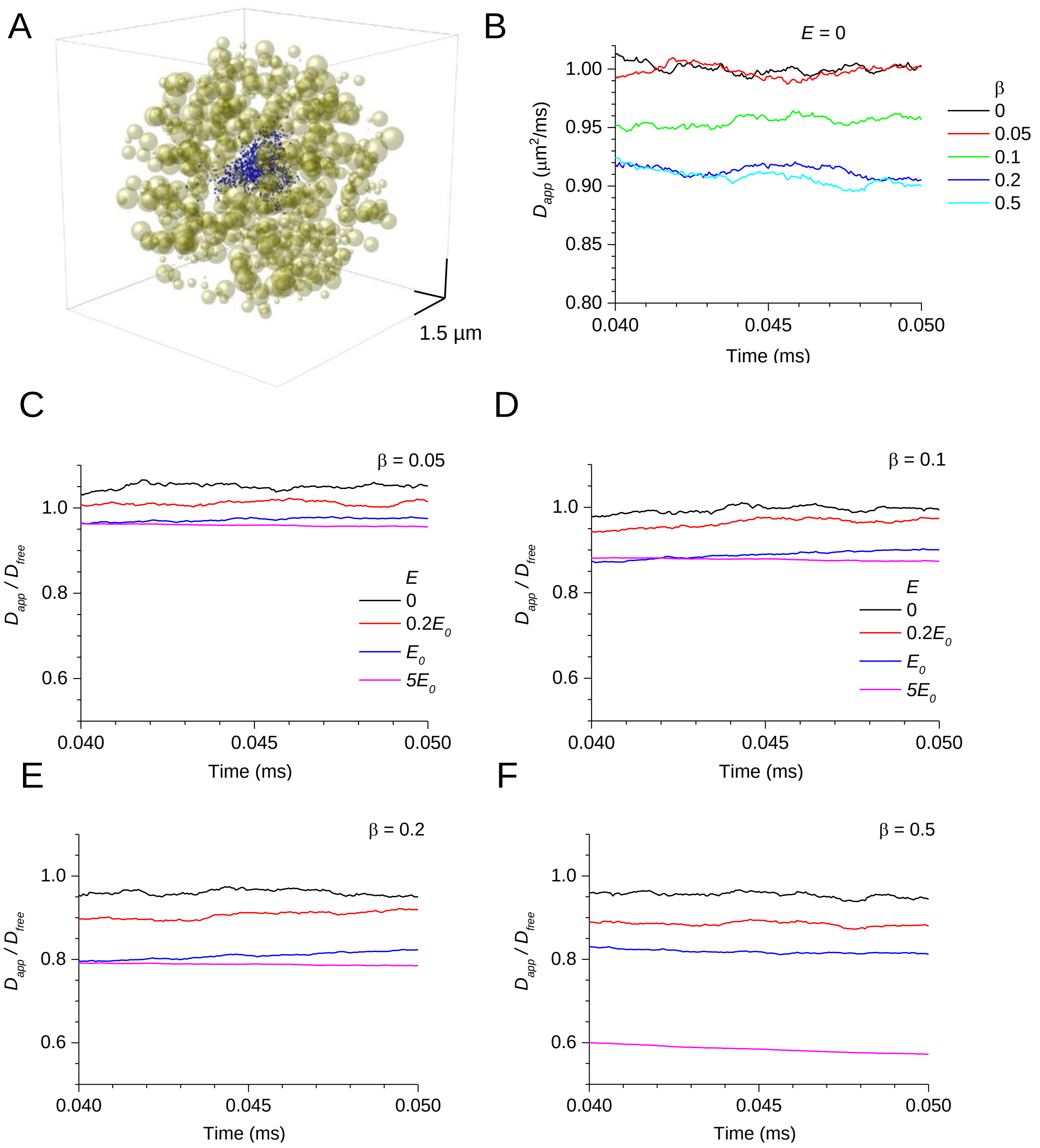

Supplementary Figure S4. Estimation of medium permeability for ions diffusing in space under 3D radial field.

(A) Simulation example: a scatter of diffusing particles as in Fig. 2D text, shown here on a larger scale for illustration purposes. Other notations are as in Fig. 2D.

(B) Example of a MC simulation run: time course of apparent diffusivity D_{app} in 3D space under E = 0.

(C-F) Examples of MC simulation runs: time course of medium permeability $\theta = D_{app} / D_{free}$, estimated for different values of β and E as indicated (same notations throughout; $E_0 = 10^4$ V/m).
